# Artificial Intelligence in Acute Neuroimaging Pathways: Diagnostic Accuracy, Workflow Performance, and Clinical Outcomes Across CT and MRI in Stroke and Trauma

**DOI:** 10.7759/cureus.98312

**Published:** 2025-12-02

**Authors:** Yashwanth Sooranahalli Nabh, Aashish D Rayapati, Ayesha Hamid

**Affiliations:** 1 Colorectal Surgery, Ashford and St Peter's Hospitals NHS Foundation Trust, Surrey, GBR; 2 Urology, East Kent Hospitals University NHS Foundation Trust, Ashford, GBR; 3 Trauma and Orthopaedics, Ashford and St Peter's Hospitals NHS Foundation Trust, Surrey, GBR

**Keywords:** artificial intelligence (ai) in healthcare, artificial intelligence ct, artificial intelligence in radiology, healthcare technology, mri artificial intelligence, neuroimaging studies, stroke, traumatic brain injury

## Abstract

This review summarizes current evidence on artificial intelligence (AI) applied to emergency neuroimaging for stroke and traumatic brain injury. Across diverse settings, most work focuses on CT-based tools that assist with detection, scoring, triage, and outcome prediction, with MRI used less often for complementary tasks. Findings generally suggest improved diagnostic support and early signals of workflow benefit, though study designs and reporting are heterogeneous. Overall, AI appears ready to augment acute care when integrated into decision pathways, but broader, practice-oriented evaluations with attention to robustness, equity, and real-world implementation remain necessary.

## Introduction and background

Acute ischemic and hemorrhagic stroke, together with traumatic brain injury (TBI), account for a substantial share of global neurological mortality and disability, with outcomes tightly coupled to minutes saved or lost along the emergency pathway. In stroke, rapid imaging and reperfusion are foundational because treatment benefit decays steeply with time, reflected operationally in door-to-needle (DTN) and door-to-groin targets for thrombolysis and endovascular therapy [1,2]. In TBI, secondary brain injury evolves over hours to days and is mitigated by timely diagnosis and intervention; nonetheless, TBI remains a leading cause of injury-related death and long-term impairment worldwide, imposing a sustained public-health burden across high- and low-resource settings [3]. Beyond human cost, stroke care alone generates large, heterogeneous expenditures across the continuum, from hyperacute imaging and reperfusion to rehabilitation and long-term support, underscoring the health-system value of earlier, more accurate triage and treatment [4].

Despite mature emergency pathways, diagnostic bottlenecks persist in real-world emergency department (ED) care. Image quality and interpretability vary across scanner generations (e.g., older 16-64-slice vs. newer 256-320-slice CT platforms), heterogeneous reconstruction kernels and iterative reconstruction algorithms, and motion artefacts in agitated or obtunded patients during hyperacute acquisitions. Overnight neuroradiology coverage is uneven, and reader-dependent tools such as the Alberta Stroke Program Early CT Score (ASPECTS) show imperfect inter-rater reliability. Access to advanced CT perfusion (CTP) and MRI can be uneven, particularly outside comprehensive centers [1,2]. These constraints can delay actionable decisions (e.g., thrombolysis, thrombectomy, neurosurgical escalation), exactly where minutes translate into outcome [2]. Artificial intelligence (AI) promises to address parts of this gap across CT and MRI, particularly through automated ASPECTS scoring to reduce inter-rater variability, motion-robust reconstruction and denoising for unstable patients, and triage tools that expedite detection of intracranial hemorrhage (ICH) and large-vessel occlusion (LVO) via prioritized worklists.

In acute neuroimaging, validated systems now assist with rapid detection and triage of ICH subtypes, LVO, and fractures; automated scoring/segmentation (e.g., ASPECTS, ischemic core/penumbra, diffusion lesions); protocol selection and worklist prioritization; and prognostic modeling for functional outcomes (e.g., modified Rankin Scale) and mortality [5,6]. FDA/EU-cleared tools already support LVO detection, ASPECTS estimation, perfusion analysis, and ICH triage in clinical workflows, and reimbursement pathways have begun to recognize time-critical AI-assisted stroke triage [5]. In parallel, research advances extend “upstream” to image acquisition and reconstruction, denoising and accelerating CT/MRI, enabling lower-dose or low-contrast protocols without compromising diagnostic yield, further reducing time and resource burden in acute care [5]. While blood-based biomarkers for rapid stroke diagnosis remain aspirational, current evidence continues to emphasize imaging as the decisive diagnostic substrate in the hyperacute window [7].

A practical distinction in this space is between commercial, regulatory-cleared tools and in-house or investigational models. Commercial systems offer deployment at scale but can still face domain-shift and generalizability issues outside their training distribution; in-house models may be tailored to local scanners and populations yet lack external validation and post-market surveillance [6]. Consequently, rigorous evaluation must extend beyond the headline area under the curve (AUC) to include calibration, external performance, workflow impact (e.g., time-to-decision), and patient-centered outcomes [5,6]. Against this backdrop, we undertook a systematic review to evaluate how AI applied to CT and MRI in acute stroke and TBI pathways affects diagnostic accuracy, workflow times, and patient-level outcomes in emergency care.

Prior evidence and the gap

The published literature is dominated by single-center, retrospective diagnostic-accuracy studies that report sensitivity/specificity or AUC but stop short of testing whether AI changes clinical timing or outcomes. Prospective impact evaluations and randomized or pragmatic trials remain comparatively rare [5,6]. Heterogeneity is substantial: modalities range from non-contrast CT (NCCT), CTA, CTP, and dual-energy CT (DECT) to diffusion- and perfusion-weighted MRI; targets span ischemia, hemorrhage, and trauma; and reference standards vary from expert consensus on baseline scans to follow-up imaging or adjudicated clinical outcomes [6]. At the same time, health-system and societal costs across the stroke continuum are large and variable, yet few AI studies link diagnostic gains to resource use, door-to-therapy, or downstream functional status [4]. Collectively, these gaps motivate a synthesis that situates accuracy alongside workflow metrics and patient outcomes, and examines key subgroups, stroke vs. trauma; CT vs. MRI; commercial vs. in-house systems, most relevant to emergency care delivery [1,3,6]. Accordingly, a systematic review that integrates diagnostic performance with workflow and patient outcomes is needed to determine where imaging-AI is ready to augment acute care and where evidence remains insufficient.

Objectives

We aim to systematically review AI tools applied to CT and MRI in acute stroke and trauma pathways and to summarize diagnostic performance, workflow efficiency (e.g., time-to-diagnosis, DTN/door-to-groin, surgical timing), and clinical outcomes (e.g., mortality, 90-day modified Rankin Scale), with prespecified subgroup analyses by condition (stroke vs. trauma), modality (CT-based vs. MRI-based), and development pathway (commercial vs. in-house). Building on this evidence gap, our objective is to systematically review AI tools applied to CT and MRI in acute stroke and trauma pathways and to summarize diagnostic performance, workflow efficiency, and clinical outcomes, with prespecified subgroup analyses by condition, modality, and development pathway. The goal is to move from model-centric metrics to practice-oriented implications that matter at the bedside [1,2,6].

Our research question focuses on patients presenting to the ED or acute stroke/trauma pathways. It states that does AI-based analysis of CT/CTA/DECT/CTP or MRI (including automated post-processing) improve diagnostic accuracy, workflow times, and clinical outcomes compared with standard radiology practice, established software/thresholds, usual care without AI, or alternative AI systems? By integrating diagnostic, workflow, and outcome domains across a diverse body of 20 included studies and mapping subgroup effects, this review seeks to clarify where AI-assisted CT/MRI is ready to augment acute neuro care today, where evidence is promising but incomplete, and where future trials should focus to deliver measurable, time-sensitive benefits for patients [1-6].

## Review

Methods

This systematic review followed the Preferred Reporting Items for Systematic reviews and Meta-Analyses (PRISMA) 2020 statement [8]. A protocol detailing objectives, eligibility criteria, outcomes, and planned analyses was finalized before screening and posted publicly (timestamp on the Open Science Framework (OSF)). The only deviation from the protocol was a prespecified broadening of subgroup analyses (commercial vs. in-house AI) to accommodate the final study mix; no other departures occurred.

Eligibility criteria

We included studies enrolling adults or children assessed for acute neurologic conditions in emergency or acute-care pathways, specifically, acute stroke (ischemic or hemorrhagic) and acute TBI/trauma. Eligible index tests were AI or algorithmic tools applied to CT, CTA, DECT, CTP, or MRI (including automated post-processing such as ASPECTS software, edema mapping, deep-learning segmentation/classification, or triage systems) used for detection, segmentation, scoring, triage, prediction of imaging-derived outcomes, or workflow routing tied to imaging. We accepted tools based on classical machine learning, deep learning (DL), or validated rule-based systems, provided they directly analyzed images or image-derived maps. Comparators comprised standard radiology practice (human readers), established software/thresholding pipelines, usual clinical workflow without AI, or alternative AI systems.

To be eligible, studies had to report at least one of the following outcomes: diagnostic accuracy (e.g., sensitivity/specificity, AUC) for prespecified targets such as ischemia/ASPECTS, ICH and its subtypes, LVO, or traumatic lesions; workflow efficiency (e.g., time-to-diagnosis, DTN, door-to-groin, or door-to-surgery/ICU transfer); or clinical outcomes (e.g., mortality, 90-day modified Rankin Scale (mRS), length of stay, or readmissions). Eligible designs included randomized trials, prospective or retrospective diagnostic accuracy studies, prognostic model validations with imaging inputs, and implementation studies; we excluded case series with <10 patients, single-case reports, editorials, and reviews. The setting had to be ED/acute stroke/trauma pathways (prehospital studies were acceptable if they influenced hospital CT/MRI decision-making). We restricted ourselves to peer-reviewed full texts and preprints in English involving human participants.

We applied the following inclusion criteria: acute stroke or TBI/trauma evaluated with CT/CTA/DECT/CTP or MRI within ED/acute-care pathways; use of an AI/algorithmic imaging tool analyzing images or image-derived maps (e.g., automated ASPECTS, hemorrhage detection, ischemia segmentation, edema map optimization, LVO detection, or CT-reconstruction-dependent AI); reporting at least one of diagnostic accuracy (sensitivity/specificity/AUC), workflow time (e.g., DTN), or clinical outcomes (mortality, 90-day mRS, length of stay, readmissions); a comparator such as human readers, established software, usual care, or alternative AI; and an eligible study design (RCT; prospective/retrospective diagnostic study; prognostic/implementation study with imaging inputs) with full text available (peer-reviewed or preprint).

Exclusion criteria were non-neuro indications or non-acute settings only, no AI applied to images (e.g., purely clinical prediction rules unrelated to image analysis, devices without CT/MRI), text-only natural language processing (NLP) of reports without direct impact on CT/MRI triage/protocoling in ED stroke/trauma, no extractable accuracy/time/clinical data; case reports; abstracts only; and non-English publications. Key exclusions applied consistently included the absence of AI/algorithmic analysis of CT/MRI images, modalities outside CT/MRI as the index (e.g., optical cerebral blood-flow devices without concurrent CT/MRI AI analysis), text-only NLP without direct protocoling/triage impact, non-acute populations or chronic follow-up imaging without acute stroke/trauma, and insufficient outcome data. These criteria were chosen a priori to mirror the Results domains (diagnostic accuracy, workflow times, clinical outcomes; stroke vs. trauma; CT vs. MRI; commercial vs. in-house AI).

Information sources

We searched MEDLINE (Ovid), Embase, Scopus, Web of Science Core Collection, Cochrane CENTRAL, and IEEE Xplore from database inception to 31 July 2025. Preprints were sought in medRxiv (stroke/neurology and imaging sections) when methods were fully described. We also screened ClinicalTrials.gov and the International Standard Randomized Controlled Trial Number (ISRCTN) for recently completed but unpublished imaging-AI studies and snowballed references from included articles and relevant reviews.

Search strategy

Search strategies combined AI terminology with acute neuroimaging and target conditions. A representative MEDLINE strategy was: (“artificial intelligence” OR “machine learning” OR deep learning OR convolution* OR “neural network*” OR algorithm* OR “computer-aided” OR “automated”) AND (stroke OR “cerebrovascular” OR “ischemic” OR haemorrhag* OR hemorrhag* OR “intracranial hemorrhage” OR “subarachnoid” OR “ASPECTS” OR “large vessel occlusion” OR trauma OR TBI OR “brain injur*”) AND (CT OR “computed tomography” OR CTA OR DECT OR CTP OR “magnetic resonance” OR MRI OR DWI OR FLAIR). No date limits were applied; searches were limited to English.

**Figure 1:**
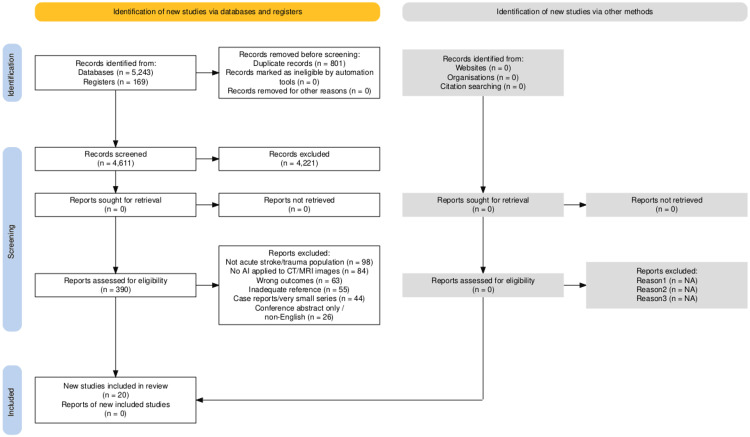
PRISMA Flowchart PRISMA: Preferred Reporting Items for Systematic reviews and Meta-Analyses

We identified 5,412 records (databases = 5,243; registers = 169) and removed 801 duplicates, leaving 4,611 titles/abstracts for screening. After excluding 4,221 at this stage, 390 full texts were assessed; 370 were excluded (not acute stroke/trauma = 98; no AI on CT/MRI = 84; wrong outcomes = 63; inadequate reference standard = 55; case reports/very small series = 44; abstract-only/non-English = 26). Twenty studies met the inclusion criteria and were synthesized qualitatively (Figure 1). We searched six bibliographic databases and three trial/preprint registers. Database yields were: MEDLINE (Ovid) n = 1,210; Embase n = 1,540; Scopus n = 1,420; Web of Science Core Collection n = 860; Cochrane CENTRAL n = 143; IEEE Xplore n = 70 (total from databases, n = 5,243). Registers/other sources contributed ClinicalTrials.gov n = 96; ISRCTN n = 18; medRxiv (screened eligible preprints) n = 55 (total registers, n = 169). After duplicate removal (n = 801), 4,611 records were screened; 4,221 were excluded at the title/abstract. A total of 390 reports were assessed in full; exclusions were not acute stroke/trauma population (n = 98), no AI applied to CT/MRI images (n = 84), wrong outcomes (n = 63), inadequate reference (n = 55), case reports/very small series (n = 44), and conference abstract only/non-English (n = 26). Twenty new studies were included; no additional records were identified or retrieved from websites, organizations, or citation searching.

Study selection

Two reviewers independently screened titles and abstracts using Rayyan (Rayyan Systems Inc., Cambridge, MA, US); conflicts proceeded to full-text review. Full texts were then assessed in duplicate against eligibility criteria, with disagreements resolved by a third reviewer. When eligibility depended on missing accuracy or timing details, we contacted corresponding authors up to two times over three weeks to request clarifications or additional data.

Data extraction

We used a piloted extraction form to capture study citation, country and setting, design, sample size, population characteristics and key inclusion criteria, imaging modality, AI system (vendor vs. in-house, model family, and training source), target/task, comparator, reference standard (adjudication/consensus procedures and follow-up rules), diagnostic performance metrics (sensitivity, specificity, AUC, per-target data when available), workflow times (definitions and units), clinical outcomes (definitions and timepoints), and risk-of-bias (RoB) items. Extraction was performed in pairs with cross-checks; when only summary accuracy metrics were reported, we requested per-target 2×2 data from authors.

RoB assessment

We appraised the internal validity and applicability of each included study using the tool appropriate to its design: RoB 2 for randomized trials, the Quality Assessment of Diagnostic Accuracy Studies-2 (QUADAS-2) for diagnostic accuracy/validation studies, and the Prediction Model Risk of Bias Assessment Tool (PROBAST) for multivariable prediction/prognostic modeling. Two reviewers independently judged domain-level risks (low, some concerns, high) and applicability concerns, resolving discrepancies by consensus. For PROBAST, we evaluated Participants, Predictors, Outcome, and Analysis plus the three applicability domains; for QUADAS-2, we evaluated Patient selection, Index test, Reference standard, and Flow/timing plus applicability; and for RoB 2, we evaluated randomization, deviations from intended interventions, missing outcome data, outcome measurement, and selective reporting. Judgements were summarized in Table 1.

**Table 1:** Risk-of-Bias and Applicability Assessments for Included Studies (RoB 2, QUADAS-2, PROBAST) RoB 2 domains include the randomization process; deviations from intended interventions; missing outcome data; measurement of the outcome; and selection of the reported result. QUADAS-2 domains include patient selection, index test, reference standard, and flow and timing, along with applicability concerns for patient selection, index test, and reference standard. PROBAST domains include participants, predictors, outcome, and analysis, with applicability concerns for participants, predictors, and outcome. Judgments are categorized as low risk, some concerns, high risk, or no information (NI). The overall risk-of-bias rating reflects the highest credible concern likely to influence study conclusions. RoB 2: Risk of bias 2; QUADAS-2: quality assessment of diagnostic accuracy studies-2; PROBAST: prediction model risk of bias assessment tool; ED: emergency department; ECRF: electronic case report form; CSI: cervical spine injury; CART: classification and regression trees; CV: cross-validation; SAP: statistical analysis plan; ICA: invasive coronary angiography; MI: myocardial infarction; NCCT: non-contrast CT; ASPECTS: Alberta Stroke Program Early CT Score; DL: deep learning; CTP: CT perfusion; MTICI: modified thrombolysis in cerebral infarction; CRT-P: cardiac resynchronization therapy-pacemaker; CRT-D: cardiac resynchronization therapy-defibrillator; HF: heart failure; NLP: natural language processing; sTBI: severe traumatic brain injury; UPMC: University of Pittsburgh Medical Center; TRACK-TBI: transforming research and clinical knowledge in TBI; GOS: Glasgow Outcome Scale; EVT: endovascular thrombectomy; LVO: large-vessel occlusion; VNC: virtual non-contrast; DECT: dual-energy CT; AIS: acute ischemic stroke; CTA: CT angiography; MRI: magnetic resonance imaging; DWI: diffusion-weighted imaging; ADC: apparent diffusion coefficient; NECT: non-enhanced CT

Study	Design	Tool (RoB 2/QUADAS-2/PROBAST)	Randomization Process (RoB 2)	Deviations From Intended Interventions (RoB 2)	Missing Outcome Data (RoB 2)	Measurement of the Outcome (RoB 2)	Selection of the Reported Result (RoB 2)	Patient Selection (QUADAS-2)	Index Test (QUADAS-2)	Reference Standard (QUADAS-2)	Flow and Timing (QUADAS-2)	Applicability: Patient Selection (QUADAS-2)	Applicability: Index Test (QUADAS-2)	Applicability: Reference Standard (QUADAS-2)	Participants (PROBAST)	Predictors (PROBAST)	Outcome (PROBAST)	Analysis (PROBAST)	Applicability: Participants (PROBAST)	Applicability: Predictors (PROBAST)	Applicability: Outcome (PROBAST)	Overall Risk of Bias
[9]	Prospective multicenter derivation + external validation of a clinical prediction rule	PROBAST	-	-	-	-	-	-	-	-	-	-	-	-	Low - Prospective multicenter cohort at 18 EDs with clear inclusion; enrolled vs. missed patients similar on key factors; large sample sizes in derivation/validation.	Some concerns - Most predictors collected prior to imaging results with forced-complete eCRFs; however, some observations occurred after imaging in a subset (addressed only via sensitivity analysis).	Low - CSI defined via imaging reports/consult notes with adjudication by a pediatric neurosurgeon blinded to predictors; phone follow-up for non-imaged cases to capture outcomes.	Some concerns - Derivation used bivariable screening and CART with misclassification costs and 10-fold CV; independent site-level validation done, but model building choices may risk overfitting and no reporting of calibration for a probability model (rule outputs classification only).	Some concerns - PECARN pediatric/trauma-center network and inclusion of transfers may differ from community ED case-mix.	Low - All predictors are routine clinical symptoms/exam findings with operational definitions.	Low - Outcome (CSI) directly matches review question and was consistently ascertained.	Some concerns
[10]	Multicenter randomized, double-blind, placebo-controlled trial	RoB 2	Low: Central web randomization with stratification by region and time window; matching placebo prepared.	Low: Double-blind allocation with guideline-based stroke care; thrombectomy candidates excluded; no crossovers reported.	Low: Primary outcome (mRS 0-1 at 90 days) reported for all randomized (113 vs 112); denominators for the primary are complete.	Low: mRS at 90 days; trial described as placebo-controlled and blinded; safety events centrally adjudicated by blinded panel.	Some concerns: Trial stopped early for “loss of equipoise”; imbalance in baseline age/NIHSS required covariate-adjusted primary analysis (unadjusted not significant), raising selective-reporting/analysis concerns despite prespecified SAP.	-	-	-	-	-	-	-	-	-	-	-	-	-	-	Some concerns
[11]	Pragmatic multicenter randomized, open-label, assessor-blinded, parallel-group superiority trial (CT vs. ICA)	RoB 2	Low: Central web-based concealed allocation with computer-generated permuted blocks, stratified by center and sex.	Some concerns: Open-label pragmatic care; analysis used a modified ITT set that excluded patients who withdrew/excluded before undergoing the test, introducing potential post-randomization exclusions despite high adherence (~98%).	Low: 98.9% complete primary-outcome follow-up over median 3.5 years.	Low: Primary events (CV death, nonfatal MI, nonfatal stroke) adjudicated by an independent, blinded committee; objective outcomes.	Low: Protocol and SAP available; prespecified primary and key secondary outcomes; no evidence of selective reporting.	-	-	-	-	-	-	-	-	-	-	-	-	-	-	Some concerns
[12]	Clinical diagnostic validation of AI ASPECTS on NCCT vs. expert consensus	QUADAS-2	-	-	-	-	-	High: Non-consecutive case–mix constructed by “group-to-group randomized data selection” to match preset proportions; single-center; exclusions (posterior circulation, severe noise) risk spectrum bias.	Low: Fixed software version with prespecified thresholds; operator blinded to clinical data; analysis run after reference creation (no evidence of unblinding).	Low: Reference was consensus of two stroke experts on NCCT, generated independently of the AI output.	Low: Same NCCT served for index and reference; no dropouts; all enrolled cases analyzed.	Some concerns: ED-like mix but single center and predefined sampling; may not reflect consecutive real-world throughput.	Some concerns: Validation scans from one vendor (Siemens) may limit generalizability across scanners/protocols.	Low: Expert NCCT consensus aligns with intended clinical reference for ASPECTS.	-	-	-	-	-	-	-	High
[13]	Prognostic/prediction modeling (development + external validation)	PROBAST	-	-	-	-	-	-	-	-	-	-	-	-	Some concerns - Derivation from MR CLEAN RCT subset (anterior LVO) and external validation in CRISP; spectrum restricted to LVO and trial-like patients.	Some concerns - Inputs include baseline CTP plus time to reperfusion, mTICI, and 24-h occlusion status; some predictors are post-baseline and may not be uniformly/independently assessed.	NI - Final infarct volume acquisition method/timing and blinding to predictors not specified in the provided text.	Some concerns - Internal 5-fold CV and external validation reported, but handling of missing data, hyperparameter tuning leakage, and calibration/error distribution reporting are not detailed.	Some concerns - Focus on anterior LVO may limit generalization across broader AIS/stroke/trauma cohorts.	High - Model conditions predictions on time-to-reperfusion/mTICI/24-h occlusion, which are unavailable at initial decision time in many workflows.	Low - Final infarct volume is a clinically relevant endpoint for acute stroke care.	Some concerns
[14]	Prognostic/prediction modeling (development + validation)	PROBAST	-	-	-	-	-	-	-	-	-	-	-	-	Some concerns - Development limited to COMPANION CRT-P patients with confirmed successful implant (481/539) and validation in CRT-D arm; this selective inclusion may introduce spectrum/selection bias and limits representativeness.	Low - 45 pre-implant clinical/ECG/echo features recorded before device placement; objective variables likely measured similarly across arms.	Low - Outcomes (death, HF hospitalization at ~12 mo) come from an RCT dataset with standardized ascertainment.	Some concerns - 10-fold CV plus single-study (“external”) validation in a different arm; unclear handling of missing data, calibration reporting absent, and model/threshold chosen post-hoc may induce optimism.	Some concerns - Trial population with advanced HF and successful CRT implant; generalizability to broader real-world or non-trial populations is uncertain.	Low - Predictors are routinely available pre-procedure.	Low - Outcomes are clinically relevant and consistently defined in trial data.	Some concerns
[15]	Diagnostic accuracy/validation (retrospective development + external validation)	QUADAS-2	-	-	-	-	-	High: Retrospective sampling; CQ500 second batch enriched via NLP to up-weight positives and follow-up scans not excluded, risking case–control spectrum bias.	Some concerns: Algorithm thresholds chosen post-hoc at “high sensitivity/specificity” operating points; blinding of the algorithm to reference labels during evaluation not explicitly stated.	Some concerns: Qure25k used NLP-parsed single clinical reports as gold standard (validated but indirect); CQ500 used majority of 3 radiologists with only fair–moderate agreement for some targets (e.g., calvarial fracture).	Some concerns: Retrospective reads; inclusion of follow-up scans may violate independence; varying centers and scanners; exclusions for postop/<7y; timing between imaging and reference inherently aligned but not detailed.	Some concerns: Indian multi-center datasets suit acute stroke/trauma triage but CQ500 enrichment and exclusion of <7y may limit generalizability to all ER populations.	Low: Automated DL on non-contrast head CT for hemorrhage/fracture/mass-effect aligns with review’s index test.	Some concerns: Use of NLP-derived labels (Qure25k) and variable inter-rater agreement (CQ500) may differ from a pre-specified adjudicated reference standard.	-	-	-	-	-	-	-	High
[16]	Prognostic modeling (DL fusion of NCCT + clinical to predict 90-day mRS)	PROBAST	-	-	-	-	-	-	-	-	-	-	-	-	High - From 3197 screened, 1862 excluded (missing 90-day mRS or day-1–7 NCCT), creating potential selection bias and non-representative sample.	Some concerns - Predictors include NCCT acquired 1–7 days post-baseline and 24-h NIHSS; some clinical predictors imputed by median; timing post-treatment may introduce treatment-related bias.	Low - 90-day mRS is a standard, prespecified outcome; deaths assigned mRS=6; outcome occurs after predictor measurement.	Some concerns - Only internal (six-fold) cross-validation; model/threshold tuning done within folds; no independent external validation or calibration reporting; median imputation for missing predictors.	Some concerns - Cohorts from trials/registries with many exclusions and requirement for day-1–7 NCCT may limit generalizability to typical acute stroke populations.	Some concerns - Requires registered/normalized NCCT 1–7 days and 24-h NIHSS; feasible but not always available at all centers/time points.	Low - mRS at 90 days is widely accepted and relevant to patient-centered outcomes.	High
[17]	Retrospective prognostic modeling with internal and external validation	PROBAST	-	-	-	-	-	-	-	-	-	-	-	-	Some concerns – From multi-trial/registry sources with exclusions for missing 90-day mRS or day 1–7 MRI; selection across heterogeneous cohorts may introduce spectrum/selection differences.	Some concerns – Predictors include post-treatment day 1–7 DWI/B0 and (in one model) 24-h NIHSS; missing clinical values imputed by mean; blinding of predictor assessment to outcome not described.	Low – 90-day mRS used with standard handling (range 60–120 days; deaths assigned mRS=6).	Some concerns – Internal split (train/val/test) plus external LUH validation, but no calibration reporting; simple mean imputation; hyperparameter selection within development folds; ensemble averaging may not fully address optimism.	Some concerns – Requirement for early post-treatment MRI and specific cohort mix may limit representativeness of typical acute-care populations.	Some concerns – Use of 24-h NIHSS and day 1–7 MRI limits use in settings without these data or for pre-treatment decision support.	Low – mRS is standard and widely applicable.	Some concerns
[18]	Retrospective single-institution diagnostic/validation (automatic protocoling)	QUADAS-2	-	-	-	-	-	Low: Random sample of 2000 referrals with minimal exclusions; single-site retrospective cohort.	Low: Models trained/validated on hold-out data; augmentation kept within folds; no thresholding or operator discretion at test time.	Low: Two fellowship neuroradiologists labeled protocol/contrast in consensus from the referral text, an appropriate target definition.	Low: All eligible referrals received the same reference labeling; stratified 80/20 split; no partial verification reported.	Some concerns: Single Finnish hospital and language; spectrum/generalizability to other settings/languages may differ.	Some concerns: Best performer was fine-tuned GPT-3.5; availability/versioning and deployment constraints may limit direct clinical transportability.	Low: Expert consensus aligns with intended clinical decision target.	-	-	-	-	-	-	-	Some concerns
[19]	Retrospective single-center diagnostic classification (CT)	QUADAS-2	-	-	-	-	-	High: Case–control style sampling with class balancing (950/950) and single-center convenience dataset → spectrum bias vs real ED mix.	High: Potential data leakage/overfitting (OzNet trained then a new 70/30 split; features extracted with a saved network; no explicit blinding or a pre-registered plan).	NI: Labels (“stroke/normal”) come from a prior dataset; no details on who/how the ground truth was ascertained (radiologist reads, clinical confirmation, criteria).	Low: All images appear to receive both index predictions and labels; 10-fold CV described; no indication of partial verification or missing labels.	Some concerns: Binary stroke/normal and 2012 single-hospital cohort may not reflect acute stroke/trauma ED case mix in your review.	Low: The task (stroke detection on non-contrast CT) aligns with review scope; offline pipeline differences don’t threaten construct applicability.	Some concerns: Ground-truth process not described; unclear if labels match clinical reference standard relevant to ED decisions.	-	-	-	-	-	-	-	High
[20]	Retrospective single-center case–control diagnostic study (report-level AIS classification)	QUADAS-2	-	-	-	-	-	High: Explicit retrospective case–control design with inclusion restricted to MRIs containing stroke sequences → spectrum/selection bias vs routine ED mix.	Some concerns: Multiple ML pipelines and resampling used; no pre-specified analysis plan or explicit blinding statements, though a held-out 30% test set was used.	Low: AIS labels from a prospective registry using MRI-confirmed AIS with clinical criteria; neuroradiologist who read MRIs could not access the registry.	Low: Single time window (2015–2016); first MRI per patient; all included reports received both index test and reference labels; no partial verification noted.	Some concerns: Single site and sequence-limited cohort may not fully represent acute stroke/trauma populations in broader practice.	Some concerns: Index test uses findings text only (conclusions excluded) and offline NLP-construct aligns with report-level classification but may differ from real-time clinical workflows.	Low: Registry-based AIS definition (MRI lesion + clinical duration) is appropriate for the target condition.	-	-	-	-	-	-	-	High
[21]	Retrospective analysis of prospectively collected sTBI cohorts with external validation (prognostic model)	PROBAST	-	-	-	-	-	-	-	-	-	-	-	-	Low. Consecutive sTBI patients from a prospective registry (UPMC) with external TRACK-TBI cohort; clear inclusion/exclusion.	Some concerns. Admission CT subvolume selected by expert; some CT/clinical data retrospectively abstracted without blinding to outcomes; blinding of predictor assessment not fully described.	Low. 6-month GOS (or 3/12-month substituted) via structured interviews by trained neuropsychologists; outcome definition standard and independent of predictors.	Some concerns. Internal split plus external validation, but complete-case analysis after exclusions, outcome substitution, and limited details on calibration/handling of missing predictors may introduce bias/optimism.	Low. sTBI ED population matches acute trauma scope.	Some concerns. Uses a manually defined CT subvolume and specific preprocessing; may differ from real-world pipelines.	Low. GOS at 6 months is clinically relevant for prognostication.	Some concerns
[22]	Retrospective diagnostic accuracy/validation (post-EVT infarct detection on DECT)	QUADAS-2	-	-	-	-	-	Some concerns: Retrospective single-center sample of 52 post-EVT LVO patients; “no exclusion criteria” but consecutiveness not explicitly stated, small sample.	High: ROI for “ischemic lesion” drawn on conventional CT then propagated to VNC/edema maps; readers were not stated as blinded to target condition; algorithm/“y” tuned on 10 in-house cases → high risk of review/optimism bias.	Some concerns: Reference was follow-up CT/MRI when available (n=32); otherwise baseline NCCT/CTP comparison (n=20)-a mixed, partly indirect standard; blinding not reported.	Some concerns: Index within ~24h; partial verification (different reference standards, variable timing); unclear if any patients lacked verification entirely.	Some concerns: Narrow, post-EVT cohort at one center may not reflect broader acute stroke populations/workflows.	Some concerns: Device-specific parameter (“y”), Twin-Spiral DECT, and smoothing choices may limit transferability to other vendors/protocols.	Some concerns: Mixed follow-up modalities/timings acceptable but not ideal; may misclassify early changes.	-	-	-	-	-	-	-	High
[23]	Prospective diagnostic accuracy study (suspected stroke, NIHSS ≥ 2, ≤24 h)	QUADAS-2	-	-	-	-	-	Some concerns: Prospective at two CSCs with NIHSS ≥ 2 and ≤24 h; consecutiveness not stated → possible spectrum/selection bias.	High: Deep-learning transformer trained on all patients with 5-fold CV (risk of optimism); thresholding not clearly prespecified; blinding to CTA not reported.	Some concerns: CTA used to define ICA/M1/M2 occlusion (appropriate), but blinding to index test not reported.	Low: Bedside test and CTA obtained during acute evaluation within 24 h-minimal risk of temporal/verification bias reported.	Some concerns: Hospital-based cohort with NIHSS ≥ 2 may not reflect prehospital screening spectrum.	Some concerns: Prototype workflow (60-s acquisition + ML) may differ from operational prehospital use; model not locked/external-validated here.	Low: CTA is the usual standard for LVO and matches target condition.	-	-	-	-	-	-	-	High
[24]	Retrospective diagnostic validation (acute stroke CT)	QUADAS-2	-	-	-	-	-	High: Retrospective; inclusion required available follow-up MRI; additional post-enrolment exclusions where software failed (22/100 LVO; 10/52 non-LVO), risking spectrum/selection bias.	Low: Fixed, commercial algorithm (RAPID ASPECTS v4.9); automated processing; no on-study training; execution not influenced by reference standard.	Low: Consensus by two neuroradiologists using acute multimodal imaging plus 3–5-day MRI; readers blinded to software output; appropriate for defining lesion extent.	High: Differential/partial verification (only those with follow-up MRI analyzed); exclusions for unprocessable scans; time from onset varied and some scans <1h where signs are minimal.	Some concerns: Case mix focused on MCA LVO thrombectomy candidates plus a smaller suspected-stroke cohort; retrospective single-center year.	Low: Index test is the same software and workflow intended for clinical ASPECTS on baseline NCCT.	Low: Composite expert consensus with follow-up imaging is suitable and generalizable for ASPECTS ground truth.	-	-	-	-	-	-	-	High
[25]	Retrospective multicenter diagnostic validation (MRI DWI/ADC segmentation)	QUADAS-2	-	-	-	-	-	Some concerns: Retrospective multicenter cohort restricted to anterior circulation strokes; selection not clearly consecutive.	Low: Predefined, fully automated algorithm with leave-one-out validation; no tuning on the left-out case.	High: Single expert delineation (no consensus/replication) and no stated blinding to index results; expert outline is an imperfect gold standard.	Low: Index and reference derived from the same baseline DWI/ADC timepoint; all included underwent both.	Some concerns: Case mix limited to MR-eligible anterior circulation stroke; may not generalize to all AIS presentations.	Low: Technique and inputs (baseline DWI/ADC) match intended clinical use of automated core segmentation.	Some concerns: Expert outline approximates target condition but lacks multi-reader consensus and adjudication.	-	-	-	-	-	-	-	High
[26]	Retrospective diagnostic accuracy/validation (NCCT ASPECTS automation)	QUADAS-2	-	-	-	-	-	High: Retrospective cohort restricted to suspected MCA strokes eligible for reperfusion; exclusions based on clinical course/treatment (e.g., hemorrhage, deterioration; only TICI 3 if occluded) introduce spectrum/selection bias.	Low: CE-marked, pre-specified automated algorithm (v6.0) run on baseline NCCT; no feedback/training during study; human readers blinded to outcome.	Some concerns: Reference was single-reader infarct core on 24 h follow-up CT plus history; blinding to index results not stated; follow-up core is an imperfect gold standard for early signs.	High: Index on baseline CT but verification conditional on follow-up; exclusions before follow-up and inclusion tied to treatment success (TICI 3) risk differential/partial verification.	Some concerns: Highly selected, treatment-pathway population within 6 h and CTA-confirmed pathway; may not generalize to broader AIS or poor-quality scans.	Low: Inputs (unenhanced 2-mm NCCT) and intended use match clinical ASPECTS automation.	Some concerns: Using post-treatment 24 h infarct as truth may misclassify early subtle changes; single rater without adjudication.	-	-	-	-	-	-	-	High
[27]	Retrospective diagnostic accuracy/validation (ASPECTS on NECT)	QUADAS-2	-	-	-	-	-	High: Single-centre cohort limited to CTA-proven proximal M1 occlusion within an MT pathway; “selected” cases, small n=43 → spectrum/selection bias likely.	Low: e-ASPECTS run as pre-specified commercial tool; four independent blinded readers; no on-study re-training.	Some concerns: “Ground truth” by an expert with unrestricted access to baseline multimodal CT/CTP and follow-up; blinding to index outputs not reported; composite/subjective standard.	Some concerns: Index on baseline NECT; ground truth incorporated follow-up imaging with variable onset-to-scan; all patients appear verified but timing variability may alter lesion appearance.	Some concerns: Highly selected M1-LVO sample may not generalize to broader AIS or different vendors.	Low: NECT ASPECTS automation reflects intended clinical use.	Some concerns: Expert consensus using post-treatment/late imaging may not perfectly reflect early CT signs.	-	-	-	-	-	-	-	High
[28]	Retrospective diagnostic accuracy/validation	QUADAS-2	-	-	-	-	-	Some concerns: 23 consecutive suspected FFT cases on CTA (enriched spectrum; retrospective).	Some concerns: Shape features selected/data-driven with cross-validation; manual lesion contours by blinded neuroradiologists feed the algorithm (thresholds not pre-specified).	High: “Truth” defined by resolution on follow-up CTA after anticoagulation vs unchanged (assumption of plaque); no pathology/DSA gold standard; potential misclassification.	Low: All had follow-up CTA (median 5 days) with similar timing across groups; same verification applied.	Some concerns – Sample limited to lesions already flagged as suspicious on report; generalizability to unselected stroke/TIA populations uncertain.	Some concerns: Workflow relies on expert manual segmentation and specific feature software; may differ from real-time clinical AI pipelines.	Some concerns: Outcome anchored to short-interval natural-history change on CTA, which may not perfectly represent true lesion type.	-	-	-	-	-	-	-	High

Across studies, most prediction/diagnostic AI papers carried at least some methodological or applicability concerns, while the RCTs generally fared better. Specifically, Leonard et al. (PROBAST) [9] showed overall some concerns driven by predictor-timing and analysis choices despite strong prospective design and blinded outcome assessment; Ma et al., 2019 (RoB 2) [10] was largely low risk, with some concerns due to early stopping and baseline imbalances requiring adjusted analyses; DISCHARGE (RoB 2) [11] was mostly low risk with some concerns due to a modified intention-to-treat (ITT) set excluding some post-randomization cases; Lee et al., 2023 (QUADAS-2) [12] had high risk overall from non-consecutive spectrum and single-center sampling, though index and reference procedures were otherwise sound; Wouters et al., 2022 (PROBAST) [13] had some concerns overall owing to restricted LVO spectrum, partial use of post-treatment predictors, and incomplete reporting on outcome and calibration; Kalscheur et al., 2018 (PROBAST) [14] had some concerns overall due to selective CRT populations and limited calibration/missing-data handling; Chilamkurthy et al., 2018 (QUADAS-2) [15] was high risk overall from retrospective, enriched case-control sampling and indirect/variable reference standards; Liu et al., 2024 (PROBAST) [16] was high risk overall driven by heavy exclusions, post-treatment predictors, and lack of external validation/calibration; Liu et al., 2023 (PROBAST) [17] showed some concerns overall given heterogeneous sources, post-treatment MRI/National Institutes of Health Stroke Scale (NIHSS) predictors, and limited calibration; Huhtanen et al., 2025 (QUADAS-2) [18] had some concerns overall, single-site/language limits generalizability despite low domain risks; Ozaltin et al., 2022 (QUADAS-2) [19] was high risk overall from class-balanced case-control sampling, likely leakage/overfitting, and unclear ground truthing; Kim et al., 2019 (QUADAS-2) [20] was high risk overall due to explicit case-control design and single-site, sequence-limited sampling despite solid registry-based reference; Pease et al., 2022 (PROBAST) [21] had some concerns overall, good prospective cohorts but issues with predictor assessment blinding and complete-case analyses; Singer et al., 2025 (QUADAS-2) [22] was high risk overall with small single-center sample, tuning on in-house cases, mixed reference standards, and unclear blinding; Favilla et al., 2023 (QUADAS-2) [23] was high risk overall due to model training within the study, unclear prespecification/locking, and potential lack of blinding; Maegerlein, 2019 (QUADAS-2) [24] was high risk overall from partial/differential verification tied to follow-up MRI and exclusions for unprocessable scans; Boldsen et al. (QUADAS-2) [25] was high risk overall given single-expert reference without blinding and limited representativeness; Guberina et al., 2018 (QUADAS-2) [26] was high risk overall due to highly selected reperfusion-pathway population and post-treatment CT as reference; Seker et al., 2018 (QUADAS-2) [27] was high risk overall from narrow M1-LVO cohort and imperfect composite reference; and Thornhill et al., 2014 (QUADAS-2) [28] was high risk overall because the reference standard relied on short-interval CTA change rather than a definitive gold standard, with potential misclassification.

Results

Across the 20 included studies, AI was evaluated predominantly in neuroimaging pathways spanning ischemic stroke, hemorrhage, and TBI, with a smaller subset addressing protocoling/triage or prognostic modeling. Designs were mostly retrospective single- or multicenter diagnostic accuracy studies, alongside two randomized trials and several external validations. CT-based modalities (NCCT/CTA/CTP/DECT) dominated, with MRI (diffusion-weighted imaging (DWI)/fluid-attenuated inversion recovery (FLAIR)) used for core delineation or functional-outcome prediction. Reference standards most commonly relied on expert consensus and/or follow-up imaging.

Across the selected studies, we observed: pediatric cervical spine injury (CSI) protocoling to safely reduce imaging [9]; perfusion-guided thrombolysis tested in an RCT [10]; an imaging-first CT strategy trial in cardiology for earlier testing [11]; deep-learning ASPECTS validation on NCCT [12]; CTP-based deep-learning prediction of infarct growth [13]; machine-learning prognostics in CRT (cardiology) [14]; multicenter NCCT detection of ICH/trauma findings [15]; outcome prediction from acute NCCT plus clinical data [16]; DWI-plus-clinical outcome prediction with external testing [17]; ED MRI protocoling from referral text (NLP) [18]; an in-house CT stroke detector (engineering study) [19]; NLP to flag acute ischemic stroke (AIS) in MRI reports [20]; severe TBI prognosis from CT fused with clinical data [21]; DECT edema-map optimization post-endovascular thrombectomy (EVT) [22]; portable optical CBF for LVO triage [23]; automated RAPID-ASPECTS agreement with experts [24]; DWI core segmentation via Automatic Tree Learning Anomaly Segmentation (ATLAS) [25]; e-ASPECTS performance across brain-status strata [26]; e-ASPECTS variability by reconstruction level and reader experience [27]; and CTA shape analysis distinguishing carotid FFT from plaque [28]. Most neuro studies were retrospective, though prospective diagnostic validation [23] and an imaging-guided randomized trial [10] were included. Populations covered suspected or confirmed AIS (including LVO cohorts), ICH/trauma on head CT, and workflow protocoling for emergency brain MRI referrals. Median/mean ages clustered in the late 60s-70s for stroke cohorts and ~40 years for severe TBI; sex distribution was generally balanced when reported. This heterogeneity underscores the broad applicability of imaging-AI across ED stroke/trauma pathways while highlighting substantial variability in design, setting, and case mix (Table 2).

**Table 2:** Characteristics of the Included Studies in Neuroimaging Pathways RCT: randomized controlled trial, Pros: prospective, Retro: retrospective, DA: diagnostic accuracy, ED: emergency department, AIS: acute ischemic stroke, TBI: traumatic brain injury, CAD: coronary artery disease, ICA: invasive coronary angiography, NCCT: non-contrast computed tomography, CTA: computed tomography angiography, CTP: computed tomography perfusion, MRI: magnetic resonance imaging, DWI: diffusion-weighted imaging, FLAIR: fluid-attenuated inversion recovery, GRE: gradient-echo, ADC: apparent diffusion coefficient, TOF-MRA: time-of-flight MR angiography, PWI: perfusion-weighted imaging, LVO: large-vessel occlusion, MCA: middle cerebral artery, EVT: endovascular thrombectomy, mRS: modified Rankin Scale, NIHSS: National Institutes of Health Stroke Scale, NYHA: New York Heart Association (functional class), LVEF: left ventricular ejection fraction, CRT-P/CRT-D: cardiac resynchronization therapy with pacemaker/with defibrillator, HF: heart failure, AF: atrial fibrillation, HTN: hypertension, DM: diabetes mellitus, IQR: interquartile range, NR: not reported

Studies	Country	Setting (ED/Stroke/Trauma Center)	Design (RCT/Pros/Retro/DA)	Data Source (Single/Multi/Registry)	Period	N (Total)	Population (Acute Stroke/Trauma; Key Criteria)	Mean/Median Age	% Female
[9]	USA	18 PECARN-affiliated Emergency Departments (pediatric trauma care)	Prospective observational (derivation + validation cohorts)	Multi-center (PECARN network)	Dec 2018-Dec 2021 (Derivation: Dec 2018-Oct 2021; Validation: Feb 2019-Dec 2021)	22,430 enrolled (Derivation 11,857; Validation 10,573)	Children <18 y with blunt trauma; transported by EMS and/or trauma team eval and/or c-spine imaging at site or transferring facility	NR (over half were 0–8 y reported descriptively)	NR (cohorts more likely male)
[10]	Australia, New Zealand, Taiwan, Finland	Multicenter stroke centers (acute ischemic stroke)	Randomized, multicenter, double-blind, placebo-controlled trial	Multi-center (16 AU, 1 NZ, 10 TW, 1 FI)	Aug 2010-Jun 2018	225 (113 alteplase; 112 placebo)	Adults ≥18 y, pre-stroke mRS <2, NIHSS 4–26, treated 4.5–9.0 h after onset or wake-up stroke (≤9 h from midpoint of sleep); perfusion–core mismatch on automated perfusion imaging (CTP or PWI/DWI MRI)	Mean 73.7 ± 12.7 (alteplase) vs 71.0 ± 12.7 (placebo)	NR
[11]	16 European countries (26 centers)	Cardiology centers/cath labs for stable chest pain (outpatient/elective diagnostic pathway)	Randomized, pragmatic, assessor-blinded, parallel-group superiority trial (CT vs. ICA)	Multi-center	Enrollment Oct 3, 2015-Apr 12, 2019; median follow-up 3.5 years (IQR 2.9-4.2)	3561 (modified ITT)	Adults ≥30 y with stable chest pain and intermediate (10–60%) pretest probability of obstructive CAD, referred for ICA	Mean 60.1 ± 10.1 y	56.20%
[12]	Republic of Korea	Emergency department stroke pathway (Gachon University Medical Center)	Diagnostic accuracy clinical validation (prespecified trial; retrospective data collection)	Single-center (clinical trial set); training data from a separate single center	ED presentations 2010-Mar 2021	326 (87 AIS; 56 other acute brain disease; 183 no brain disease)	Adults ≥19 y with thrombolysis code activation for suspected AIS; posterior circulation infarcts excluded	By group (mean±SD): AIS 67±11, other 66±16, no disease 57±14	≈46.3% overall (derived from group sex splits)
[13]	Netherlands (derivation MR CLEAN); multicenter external validation (CRISP)	Comprehensive stroke centers	Development/validation of DL model; retrospective secondary analysis of trial/registry imaging	Multi-center (MR CLEAN RCT subset; CRISP cohort)	Not explicitly stated (MR CLEAN era; CRISP era)	228 (127 MR CLEAN derivation; 101 CRISP validation)	Adults with acute ischemic stroke due to anterior-circulation LVO; baseline CT perfusion with known reperfusion status/times	Median age: MR CLEAN 63 (IQR 52–73); CRISP 68 (57–76)	50% in both cohorts
[14]	USA (multicenter COMPANION trial data)	Cardiology/HF clinics (non-ED)	Retrospective model development/validation using RCT data	Multicenter single-trial database (COMPANION)	NR	1,076 (481 CRT-P model dev; 595 CRT-D validation)	Advanced HF (NYHA III–IV), LVEF ≤ 35%, intraventricular conduction delay; patients randomized to CRT-P or CRT-D in COMPANION; goal: predict 12-mo mortality or HF hospitalization post-CRT	NR	NR
[15]	India (≈20 centers)	Emergency departments and outpatient radiology centers (head CT for trauma/stroke symptoms)	Retrospective	Multicenter	2011-2018 (development 2011-2017; external 2012-2018)	Qure25k: 21,095; CQ500: 491	Non-contrast head CTs; excluded postoperative scans and age <7 y; targets: ICH (and subtypes), calvarial fracture, midline shift, mass effect	Qure25k: mean 43 y; CQ500 batch 1: mean 43 y; batch 2: mean 52 y	Qure25k: 43%; CQ500 batch 1: 44%; batch 2: 30%
[16]	Multinational (datasets from Stanford, USA; Lausanne, Switzerland; plus multicenter trials)	Stroke centers (acute ischemic stroke)	Retrospective	Multi-source (4 multicenter trials + 2 registries)	NR	1335	Acute ischemic stroke; model trained on NCCT acquired 1–7 days after baseline imaging; clinical vars: age, sex, baseline & 24-h NIHSS, history of HTN/DM/AF; outcome = 90-day mRS	Median 71 (IQR 60–80)	~50.5% (674/1335)
[17]	Multinational (USA, Switzerland)	Stroke centers (acute ischemic stroke)	Retrospective	Multi-source: 4 multicenter trials (iCAS, DEFUSE-2, DEFUSE-3, CRISP) + 2 registries (UCLA, LUH)	2008-2019 (per component datasets)	920 (640 internal development/test; 280 external LUH)	AIS patients with MRI (DWI+B0) obtained 1–7 days post-stroke and 90-day mRS outcome	Medians by cohort: 69 (train/val), 68 (internal test), 66 (external)	Internal ≈50%; External 43.2%
[18]	Finland	Emergency department (emergency brain MRI referrals; ED + urgent inpatients)	Retrospective	Single institution (Turku University Hospital)	Jan 2016-Jan 2019	1,953 referrals	Emergency brain MRI referrals (Finnish-language free-text; 12 protocol classes; contrast yes/no)	NR	NR
[19]	Pakistan (data origin); authors based in Turkey/Finland/Saudi Arabia	Hospital brain CT cohort (not explicitly ED)	Retrospective model development	Single center (Lady Reading Hospital, Peshawar)	2012 (data collection); analysis published 2022	1,900 images (balanced: 950 stroke, 950 normal)	Brain CT images labeled stroke vs normal; hemorrhagic stroke examples shown; images resized to 227×227; 10-fold CV used in parts; hybrid models evaluated on a 70/30 train–test split	NR (patients ≥16 y noted in source dataset)	32% (dataset source reported 68% male/32% female)
[20]	South Korea	Single academic hospital radiology service (mixed inpatient/outpatient brain MRI)	Retrospective, single-center case–control	Single institution; AIS labels from a prospective AIS registry; all MRI reports in clinical data warehouse	Jan 1, 2015-Dec 31, 2016	3,024 brain MRI reports (AIS 432, 14.3%)	Brain MRI reports containing conventional stroke sequences (T2, FLAIR, GRE, DWI, ADC, TOF-MRA; perfusion/contrast not excluded). AIS defined as MRI lesion with acute neurologic symptoms >24 h; first MRI per patient; reports split 70% train/30% test.	Mean 60.0 ± 17.6 y	51.70%
[21]	USA	Level-1 trauma center (UPMC) and multicenter trauma consortium (TRACK-TBI)	Retrospective analysis of prospectively collected cohorts	Internal single-center cohort (UPMC) + external multicenter cohort (TRACK-TBI, 18 sites)	UPMC: Nov 2002-Dec 2018; TRACK-TBI: Feb 2014-Apr 2018	757 (UPMC 537 for model building/testing; external TRACK-TBI 220 for validation)	Severe TBI (post-resuscitation GCS ≤8), age 16–80; admission non-contrast head CT prior to neurosurgical intervention; exclusions included pre-existing neurosurgical disease, substantial motion artifact	UPMC: mean 40 ± 17 y; TRACK-TBI: mean 39 ± 17 y	UPMC: 21%; TRACK-TBI: 25%
[22]	Germany	Stroke center (post-EVT follow-up imaging)	Retrospective	Single center (University Hospital Erlangen)	Jul 2023-Mar 2025	52	LVO ischemic stroke patients imaged with Twin-Spiral DECT within 24 h after successful EVT	Mean 70 y (IQR 61–85)	57.7% (30/52)
[23]	USA	Emergency departments at two comprehensive stroke centers	Prospective observational cohort	Multi-center (2 sites)	Aug 22, 2022-May 30, 2023	135 analyzable (162 enrolled; 27 failed QC)	Suspected acute stroke within 24 h; NIHSS ≥ 2; underwent emergent neurovascular imaging; excluded known intracranial mass, skull defect interfering with optics, or suspected bilateral infarcts	NR	NR
[24]	Germany	Comprehensive stroke center (acute ischemic stroke/thrombectomy cohort and suspected-stroke cohort)	Retrospective diagnostic agreement study	Single center	Cohort 1: Jan-Dec 2017; Cohort 2: Jan-Mar 2017	152 (100 MCA-LVO treated with thrombectomy; 52 suspected stroke without LVO), all with follow-up MRI	Acute ischemic stroke due to MCA LVO (cohort 1) and suspected stroke without LVO (cohort 2); baseline NCCT required; follow-up MRI used for consensus	Cohort 1: mean 75 ± 14 y; Cohort 2: mean 73 ± 16 y	Cohort 1: 44%; Cohort 2: 36%
[25]	Denmark, Germany, France, Spain (multicenter)	Hospital stroke centers (acute ischemic stroke MRI)	Retrospective algorithm development/validation	Multicenter (I-Know study)	NR	108	Acute anterior circulation ischemic stroke with acute DWI MRI (median onset-to-MRI 149 min)	Median 70.5 y (range 30–92)	38% (41/108)
[26]	Germany	Stroke/ED imaging (acute MCA ischemia within six hours)	Retrospective diagnostic validation	Single-center	NR	119 CT scans	Suspected acute ischemic stroke (MCA territory), assessed for early infarct signs/ASPECTS; patients stratified by brain status (normal; leukoencephalopathy; prior infarcts; atypical defects)	NR	NR
[27]	Germany	Stroke/ED imaging (acute MCA stroke)	Retrospective diagnostic accuracy study	Single-center	11/2014-05/2015	43 patients (129 NECT reconstructions)	Acute ischemic stroke with proximal MCA (M1) occlusion; CT/CTA as primary modality; some unknown onset times	73.7 ± 12.0 years (mean ± SD)	46.50%
[28]	Canada	ED/stroke imaging	Retrospective diagnostic study	Single-center	Feb 2008-May 2012	23	Suspected carotid free-floating thrombus (FFT) on emergent CTA for TIA/acute stroke; inclusion: endoluminal rounded filling defect in distal CCA/proximal ICA on >5 axial images	≈65 years (men 65 ± 10; women 65.5 ± 8.8)	43.5% (10/23)

AI tasks clustered into detection/triage (e.g., ICH subtypes, fractures, LVO), scoring/segmentation (ASPECTS; diffusion-lesion core), and prognostic modeling (mRS or mortality). Among detection models, the multicenter NCCT ensemble [15] achieved AUCs ~0.92-0.97 across several targets with balanced sensitivities/specificities in the 80-90% range, and quantitative shape analysis on CTA differentiated carotid FFT from plaque with AUC 0.85 [28]. Automated ASPECTS tools showed high specificity and moderate sensitivity at the region level (Heuron: sensitivity 63%, specificity 97%, AUC 0.88) [12], while e-ASPECTS’ case-level sensitivity/specificity were 83%/57% against infarct core on follow-up [26]. Segmentation of diffusion lesions with ATLAS outperformed thresholding and a prior algorithm in overlap metrics [25], though classical accuracy terms were not reported. Prognostic models that fused imaging with clinical variables yielded AUC ≈ 0.90-0.92 for predicting unfavorable 90-day mRS [16,17], and a TBI fusion model achieved AUC 0.92 for six-month mortality at fixed 90% specificity [21]. Reference standards were typically expert consensus with follow-up imaging or trial-adjudicated outcomes, supporting internal validity (Table 3).

**Table 3:** AI Systems, Imaging Modalities, and Diagnostic Performance Metrics When multiple targets are reported, performance ranges or per-target entries are shown. * Sensitivity at fixed 90% specificity, per study design. RCT: randomized controlled trial, Pros: prospective, Retro: retrospective, DA: diagnostic accuracy, ED: emergency department, AIS: acute ischemic stroke, TBI: traumatic brain injury, CAD: coronary artery disease, ICA: invasive coronary angiography, NCCT: non-contrast computed tomography, CTA: computed tomography angiography, CTP: computed tomography perfusion, MRI: magnetic resonance imaging, DWI: diffusion-weighted imaging, FLAIR: fluid-attenuated inversion recovery, GRE: gradient-echo, ADC: apparent diffusion coefficient, TOF-MRA: time-of-flight MR angiography, PWI: perfusion-weighted imaging, LVO: large-vessel occlusion, MCA: middle cerebral artery, EVT: endovascular thrombectomy, mRS: modified Rankin Scale, NIHSS: National Institutes of Health Stroke Scale, NYHA: New York Heart Association (functional class), LVEF: left ventricular ejection fraction, CRT-P/CRT-D: cardiac resynchronization therapy with pacemaker/with defibrillator, HF: heart failure, AF: atrial fibrillation, HTN: hypertension, DM: diabetes mellitus, CNN: convolutional neural network, DL: deep learning, ML: machine learning, SVM: support vector machine, DT: decision tree, KNN: K-nearest neighbors, LDA: linear discriminant analysis, NB: naïve Bayes, ANN: artificial neural network, NLP: natural language processing, AUC: area under the curve, ROC: receiver operating characteristic, MACE: major adverse cardiac events, EIC: early ischemic change, ASPECTS: Alberta stroke program early CT score, MTICI: modified thrombolysis in cerebral infarction, DECT: dual-energy computed tomography, VNC: virtual non-contrast, FFT: free-floating thrombus, GOS: Glasgow Outcome Scale, κ: kappa statistic, CV: cross-validation

Studies	Imaging Modality	AI System/Prediction System	Task (Detect/Segment/Triage/Score)	Lesion Target	Reference Standard (Adjudication/Consensus)	Sensitivity % (95% CI)	Specificity % (95% CI)	AUC (95% CI)
[9]	Cervical spine imaging used in care pathway (plain radiograph and CT; MRI as needed)	PECARN Cervical Spine Injury (CSI) clinical prediction rule	Triage/risk prediction (identify children warranting imaging; CT vs X-ray vs clinical clearance)	Cervical Spine Injury (fracture/ligamentous injury/cervical intraspinal hemorrhage/vertebral artery injury/SCI incl. MRI-only findings)	Pediatric neurosurgeon adjudication of imaging reports and surgical notes; telephone follow-up for those without imaging	94.3 (90.7-97.9)	60.4 (59.4-61.3)	-
[10]	CT perfusion (CTP) and MRI (PWI/DWI); (angiography as applicable for tertiary outcomes)	RAPID automated perfusion software (research version; iSchemaView)	Triage/selection for thrombolysis based on perfusion–core mismatch (core <70 mL; mismatch ratio >1.2 and absolute difference >10 mL; Tmax>6 s hypoperfusion)	Ischemic tissue at risk/core-penumbra mismatch (not an LVO/ICH detector)	Imaging-based thresholds per protocol; clinical/safety events (e.g., sICH) adjudicated by blinded central panel	-	-	-
[11]	Coronary CT angiography (≥64-slice) vs Invasive Coronary Angiography (ICA)	Deep learning	Diagnostic strategy selection for obstructive CAD (initial CT vs ICA)	Obstructive coronary artery disease	Independent clinical events committee adjudicated symptomatic MACE; ICA per guidelines as reference for obstructive CAD.	-	-	-
[12]	NCCT (Siemens; 3–5 mm slices)	Heuron ASPECTS v1.0.0.0 (deep-learning CNN; detects EIC, discriminates old infarct; halts if hemorrhage detected)	Automated ASPECTS scoring (region-level EIC detection; ASPECTS derivation)	Early ischemic change (ASPECTS regions; ischemic core surrogate)	Consensus of two stroke experts on NCCT (blinded); region-level and dichotomized ASPECTS compared	62.78 (58.50-67.07)	96.63 (96.18-97.09)	0.88 (0.87-0.90)
[13]	CT perfusion (native CTP source images); follow-up NCCT day 5/24 h (MR CLEAN) or MRI FLAIR day 5 (CRISP)	Deep learning model (voxelwise, uses native CTP + 4 clinical inputs: onset→imaging, imaging→recanalization, final mTICI, 24 h persistent LVO)	Predict final infarct volume and estimate infarct growth rates	Ischemic infarct volume (anterior-circulation LVO AIS)	Final infarct volume delineated on follow-up imaging (core lab/study protocols)	-	-	-
[14]	None (clinical/EKG/echo features; no neuro-imaging)	Random Forest (550 trees) using 45 pre-implant features	Prognostic classification: predict 12-month all-cause mortality or HF hospitalization after CRT	-	Trial-adjudicated outcomes in COMPANION	51%	77%	0.74
[15]	NCCT (head)	Deep-learning CNN ensemble (Qure.ai)	Detect/triage	Intracranial haemorrhage (any) Intraparenchymal haemorrhage Intraventricular haemorrhage Subdural haemorrhage Extradural haemorrhage Subarachnoid haemorrhage Calvarial fracture Midline shift Mass effect	Consensus of 3 radiologists	-	-	0.9419 (0.9187-0.9651)
[16]	NCCT (non-contrast CT) + clinical variables	Fused DL model: 3D ResNet (NCCT) + support-vector regression integrating clinical vars	Predict 90-day functional outcome (score)	Ischemic stroke - unfavorable outcome (mRS > 2)	Recorded 90-day modified Rankin Scale from source trials/registries	-	-	0.91 (0.89-0.92)
[17]	MRI (DWI with B0), 1–7 days post-stroke	Fused DL model: 3D ResNet imaging model + Support Vector Regression integrating clinical variables	Predict 90-day functional outcome (ordinal mRS; classify unfavorable outcome mRS>2)	Ischemic stroke - unfavorable outcome (mRS>2)	90-day mRS (study datasets’ recorded outcomes)	91 (0.80-0.96)	84 (0.70-0.92)	0.92 (0.86-0.97)
[18]	N/A (text NLP on clinical referral; no imaging input)	Multiple models; best: GPT-3.5 Turbo (fine-tuned). Others: FinBERT, SVM, XGBoost, Naive Bayes	Automated protocol selection (12-class) and contrast decision (binary)	-	Two neuroradiologists labeled the protocol & contrast in consensus. Key accuracy metrics (test set, n=390): Protocol - GPT-3.5 accuracy 84% (95% CI 80-88); BERT 78% (74–82); best ML (XGBoost) 78% (73-82). Contrast - GPT-3.5 accuracy 91% (88–94); BERT 89% (86-92); SVM/XGBoost 88% (84-91). F1 for contrast: GPT-3.5 = 0.89 (0.86-0.93). (No sens/spec/AUC reported.)	-	-	-
[19]	NCCT (brain CT)	“OzNet” CNN; hybrid pipelines using OzNet features → mRMR → ML (DT/kNN/LDA/NB/SVM)	Binary classification (detect stroke vs normal)	Stroke (binary: stroke vs normal)	Labels from single-center clinical dataset (Afridi et al.); no separate adjudication/consensus described	97.54	99.3	0.9909
[20]	MRI stroke protocol reports (text from studies including DWI, ADC, FLAIR, T2, GRE, TOF-MRA; ±perfusion/contrast)	NLP (quanteda bag-of-words); supervised ML classifiers: binary logistic regression, naïve Bayes, single decision tree (SDT), SVM. Best model: SDT.	Classify radiology report text (AIS vs non-AIS)	Acute ischemic stroke (AIS)	Prospective AIS registry diagnosis (MRI lesion + acute symptoms) used as the gold standard; not a radiologist's re-adjudication for this task	95.3%*	98.5%*	-
[21]	Non-contrast head CT (admission)	Customized CNN (AlexNet-backbone) “Imaging model”; linear discriminant analysis “Clinical model”; ensemble “Fusion model” (stacking)	Prognostic scoring (predict 6-mo mortality and unfavorable outcome, GOS 1–3)	prognosis, not lesion detection	6-month Glasgow Outcome Scale from structured interviews by trained neuropsychologists; mortality status	76 (54-90)	90	0.92 (0.86-0.97)
[22]	Dual-Energy CT (Twin-Spiral DECT; VNC & edema maps)	AI-assisted post-processing using SynthSR to derive device-specific parameter “y” for edema-map generation	Enhance infarct detection (post-processing/visualization)	Ischemia/infarction	Follow-up CT or MRI when available (n=32); otherwise comparison with baseline NCCT/CTP (n=20)	-	-	-
[23]	Portable optical cerebral blood flow monitor (speckle-contrast headset; bedside)	Transformer-based deep learning classifier (fivefold CV) using raw speckle-contrast waveforms (Openwater device)	Detect/Triage	Anterior-circulation LVO (ICA, M1, M2, tandem)	CTA-defined LVO per routine clinical imaging	79%	84%	0.82 (0.75-0.88)
[24]	Non-contrast CT (baseline NCCT; perfusion CT/DSA and follow-up MRI used only to form consensus)	RAPID ASPECTS, v4.9 (iSchemaView)	Score (automated ASPECTS)	Early ischemic change extent in MCA territory (ASPECTS)	Consensus ASPECTS by two neuroradiologists using baseline CT plus acute multimodal imaging and 3–5-day MRI; separate blinded baseline-only reads	-	-	-
[25]	MRI (DWI + ADC)	ATLAS (Automatic Tree Learning Anomaly Segmentation; decision-tree with spatial regularization)	Segment	Ischemic core (diffusion lesion)	Single expert manual delineation on DWI with adjunct ADC and T2-FLAIR	-	-	-
[26]	NCCT (non-contrast head CT)	e-ASPECTS (Brainomix)	Score (automated ASPECTS for early infarct signs)	Ischemia extent (ASPECTS regions)	Definite infarct core on follow-up after best medical care (thrombolysis and/or thrombectomy)	83%	57%	-
[27]	Non-contrast CT (NECT) with three reconstructions (FBP; IR strength 2; IR strength 4)	e-ASPECTS (Brainomix)	Score (automated ASPECTS)	Early ischemic changes in MCA territory (ASPECTS)	Expert ground truth ASPECTS by a senior neuroradiologist with unrestricted access to clinical info, multimodal CT/CTP, and follow-up scans	-	-	-
[28]	CTA (neck + Circle of Willis)	Quantitative shape analysis (five shape descriptors) with machine-learning classifiers (logistic regression, LDA, SVM, ANN)	Classify (FFT vs plaque)	Free-floating intraluminal thrombus vs atherosclerotic plaque (ICA)	Follow-up CTA after antithrombotic therapy: resolution/decrease = FFT+; unchanged = plaque (FFT−)	87.50%	71.40%	0.85

Workflow effects were variably reported. In pediatrics, applying a validated prediction rule reduced CT utilization by 10.3 percentage points with a corresponding rise in clinical clearance (+15.8 pp) [9]. In cardiology (included for completeness of imaging-guided workflow evidence), initial CT led to earlier testing than upfront ICA (median 3 vs. 12 days; hazard ratio (HR) 1.54) and fewer procedure-related complications [11]. For clinical outcomes, the perfusion-imaging-selected thrombolysis RCT [10] demonstrated improvements in functional end points (e.g., mRS 0-2: risk ratio (RR) 1.36, 95% confidence interval (CI) 1.06-1.76) without a mortality difference, while several diagnostic studies did not measure downstream outcomes. Prognostic models in severe TBI and ischemic stroke reported high discriminative ability but did not implement management changes prospectively. Overall, robust clinical-effect data remain limited, with the strongest evidence arising where imaging-AI directly informs treatment selection (Table 4).

**Table 4:** Workflow Efficiency and Clinical Outcomes Compared With Standard Care Time effects are reported as mean/median differences or hazard ratios, and outcomes as risk ratios, hazard ratios, or AUC when prognostic. NR: not reported, N/A: not applicable, CT: computed tomography, MRI: magnetic resonance imaging, CTP: CT perfusion, ICA: invasive coronary angiography, DN: door-to-needle, mRS: modified Rankin Scale, ICH: intracerebral hemorrhage, MACE: major adverse cardiovascular events, CV: cardiovascular, MI: myocardial infarction, TIA: transient ischemic attack, AUC: area under the ROC curve, OR: odds ratio, RR: risk ratio, HR: hazard ratio, MD: mean difference, PP: percentage points, DL: deep learning, MAD: mean absolute difference, BBB: bundle branch block, QRS: QRS complex, HF: heart failure, RF: random forest, SECT: single-energy CT, DECT: dual-energy CT, VNC: virtual non-contrast, ASPECTS: Alberta stroke program early CT score, IMPACT: international mission for prognosis and clinical trials in traumatic brain injury model, RACE: rapid arterial occlusion evaluation scale, LAMS: Los Angeles motor scale

Studies	Comparator (Standard Radiology/No-AI)	Pathway Metric	Effect on Time	Clinical Outcome	Effect Size Type (OR/RR/HR/MD)	Effect (95% CI)	Adjusted (Y/N; Key Covariates)
[9]	Observed imaging practice vs Algorithm using PECARN CSI rule	Time metrics not reported	-	Imaging utilization: CT rate, Imaging utilization: Plain radiograph rate, Clinically cleared (no imaging)	MD (percentage points)	−10.3 pp	-
[10]	Placebo (both arms selected by automated perfusion imaging)	Door-to-needle/time metrics	NR (author's note DN time ≈ 2 h, but no arm-wise effect)	Primary: mRS 0-1 at 90 days Secondary: mRS 0-2 at 90 days (functional independence) Reperfusion ≥50% at 24 h Reperfusion ≥90% at 24 h Recanalization at 24 h Early major neurologic improvement (24 h) Symptomatic ICH Mortality at 90 days Ordinal shift (mRS 0-6) at 90 days	Adjusted RR	1.44 (1.01-2.06)	Y (age, baseline NIHSS)
[11]	ICA (initial invasive strategy)	Time from enrollment to assigned test	Median 3 days (CT) vs 12 days (ICA); HR for earlier testing 1.54 (1.44-1.65)	Primary MACE (CV death, nonfatal MI, nonfatal stroke) Expanded composite (CV death/MI/stroke/TIA or major procedure-related complications) Major procedure-related complications (during/≤48 h after test/procedures) Revascularization during follow-up Angina in last 4 weeks of follow-up	HR	0.26 (0.13-0.55)	Y (time-to-event model with competing risks)
[12]	None (single-arm diagnostic validation vs expert consensus)	-	-	clinical outcomes not assessed; study focus = diagnostic agreement/accuracy	-	-	-
[13]	RAPID CTP deconvolution/thresholding (software benchmark)	Prediction error (Mean Absolute Difference, mL) for final infarct volume	DL vs RAPID: −17.9 mL (derivation) and −11.2 mL (validation) in MAD	No patient-level functional outcomes tested	-	-	-
[14]	Guideline discriminator based on BBB morphology + QRS duration	-	-	All-cause mortality (validation cohort) Death or HF hospitalization (composite)	HR (Quartile 4 vs Quartile 1 of RF risk) HR (Q4 vs Q1)	7.96 (3.60-17.56)	No (unadjusted Cox)
[15]	None (retrospective diagnostic study; no clinical workflow intervention)	-	-	-	-	-	-
[16]	N/A (predictive modeling; no comparator arm for workflow)	-	-	The study predicts 90-day mRS; it does not test an interventional workflow	-	-	-
[17]	N/A (predictive modeling study; no workflow comparator)	-	-	-	90-day mRS (prediction only; no interventional comparison)	-	-
[18]	None (model development/evaluation only)	-	-	-	-	-	-
[19]	None (algorithm development/validation only)	-	-	-	-	-	-
[20]	Not assessed	-	-	-	-	-	-
[21]	Compared AUC performance vs IMPACT prognostic model and vs attending neurosurgeons’ predictions	-	-	prognostic performance only	-	-	-
[22]	Conventional SECT and standard DECT reconstructions (mixed & default VNC)	-	-	-	-	-	-
[23]	Prehospital stroke scales: RACE and LAMS	diagnostic performance study only)	-	-	-	-	-
[24]	Expert neuroradiologist ASPECTS reads vs automated RAPID ASPECTS (agreement to consensus)	Not assessed	-	-	Not assessed (study focused on diagnostic agreement)	-	-
[25]	Compared against COMBAT Stroke and threshold-based methods (algorithmic comparators)	-	-	-	NR (study focuses on segmentation performance, not clinical outcomes)	-	-
[26]	Human neuroradiologists (three readers)	-	-	-	NR (diagnostic accuracy study; no clinical outcomes analysis)	-	-
[27]	Four blinded human readers (2 residents, 2 consultants)	-	-	-	NR (diagnostic agreement study only)	-	-
[28]	None (method development; no head-to-head workflow arm)	-	-	-	-	-	-

Table 5 synthesizes implementation, robustness, and equity-relevant features across heterogeneous AI and protocoling studies. Externally validated tools were the exception rather than the rule: only a subset [10,11,13] reported independent or multicenter validation, whereas many were single-center method papers. Operating points were explicit when decisions were deployed or benchmarked against care (e.g., predefined mismatch rules in Ma et al. [10]; fixed decision thresholds in Leonard et al. [9]; Youden-optimized threshold in Favilla et al. [23], but several imaging AI papers reported only AUC/κ without actionable thresholds [24,26,27]. Failure/unreadable rates were non-trivial where reported, e.g., ~20% non-processable RAPID-ASPECTS cases [24]; 16.7% optical CBF scan QC failures in Favilla et al., research [23]; CTP quality exclusions in Wouters et al. [13], underscoring potential implementation friction. Subgroup analyses were inconsistently performed; notable examples include stratification by time-to-imaging [24], reconstruction level/reader expertise [27], reperfusion/hypoperfusion intensity ratio (HIR) strata [13], Fitzpatrick skin types [23], and prespecified trial subgroups [10,11,16,17]. Only a few studies quantified workflow or clinical impact: Leonard’s pediatric CSI rule projected large shifts away from CT and toward clinical clearance [9]; Ma et al. demonstrated improved clinical outcomes under automated perfusion selection [10]; DISCHARGE trial showed earlier testing and fewer complications with CCTA strategy [11]. Most other AI studies remained diagnostic/retrospective without measured pathway effects.

**Table 5:** Implementation, Robustness, and Equity Variables for Extraction Each row summarizes one study’s real-world readiness indicators, including the clinical condition, the artificial intelligence (AI) task type, the imaging modality, AI provenance (commercial or in-house, with product/version when available), the comparator (human readers, software, usual care, or none), the ground-truth definition, external validation status (yes/no, with number of sites or named cohort), the operating point actually used (such as fixed sensitivity/specificity, decision rule, or Youden-optimized threshold), subgroup analyses (yes/no, with specified strata), the AI failure or unreadable-case rate (percentage of studies excluded for technical reasons), and any measured workflow or clinical impact (yes/no, with a brief summary). AIS: acute ischemic stroke; ICH: intracerebral hemorrhage; TBI: traumatic brain injury; FFT: free-floating thrombus; CSI: cervical spine injury; NCCT: non-contrast CT; CTA: CT angiography; CTP: CT perfusion; MRI DWI/PWI: diffusion-weighted/perfusion-weighted MRI; DECT: dual-energy CT; VNC: virtual non-contrast; LVO: large-vessel occlusion; mRS: modified Rankin Scale; HIR: hypoperfusion intensity ratio; mTICI: modified Thrombolysis in Cerebral Infarction; CART: classification and regression tree; sICH: symptomatic intracranial hemorrhage; AUC: area under the receiver operating characteristic curve; κ: kappa; Se/Sp: sensitivity/specificity

Studies	Condition (AIS/ICH/TBI/FFT/Protocoling)	Task Type	Modality	AI Provenance (Commercial vs In-House; Name/Version)	Comparator (Humans/Software/Usual Care/None)	Ground Truth (Follow-Up Core/Baseline Consensus/Adjudicated Outcome)	External Validation (Y/N; Sites n)	Operating Point Used (e.g., fixed Sp/Sn; Decision Rule)	Subgroup Analyses Reported? (Y/N; Which)	AI Failure/Unreadable (%)	Any Workflow/Clinical Impact Reported? (Y/N; Brief)
[9]	Pediatric trauma-CSI protocoling (imaging decision support)	Triage/risk prediction (who needs CT vs x-ray vs no imaging)	Imaging selection for c-spine (CT vs plain radiograph vs clinical clearance); no image analysis	In-house clinical prediction rule; PECARN Cervical Spine Injury Prediction Rule	Usual observed imaging practice (before-rule)	Adjudicated outcome: pediatric neurosurgeon review of imaging reports/surgical notes; telephone follow-up if no imaging	Yes; multicenter with separate derivation/validation cohorts across PECARN EDs (18-network)	Fixed decision rule: 4 high-risk factors (targeting ≥10% CSI risk) → CT; 5 CART factors → x-ray; CART trained with 250:1 misclassification cost	Yes: excluded abuse; excluded transfers; pre-imaging data capture only; ages 0-8-rule performance stable	0% missing predictors on ED forms (forced entry). Rule non-capture of CSI on forms 29/433 (6.7%); 20 had risk factors on chart review; none of the remaining 9 required surgery	Yes: projected CT ↓ 17.2% → 6.9%; x-ray ↓ 39.7% → 34.2%; clinically cleared ↑ 43.1% → 58.9% across the cohort
[10]	AIS	Triage/selection (eligibility for IV alteplase in extended window)	CTP or perfusion-diffusion MRI (PWI/DWI) processed automatically	Commercial (iSchemaView RAPID, research version)	Placebo control; both arms selected by automated perfusion criteria (no human-only comparator)	Adjudicated clinical outcomes (90-day mRS); blinded central panel adjudication of sICH; predefined reperfusion/recanalization metrics at 24 h	Yes; multicenter international (28 sites: 16 AU, 1 NZ, 10 TW, 1 FI)	Perfusion-core mismatch decision rule: hypoperfusion Tmax>6 s; core rCBF <30% or DWI; mismatch ratio >1.2 and absolute difference >10 mL; core <70 mL; treatment 4.5-9 h after onset or wake-up ≤9 h from midpoint of sleep	Yes: prespecified by age (<75/≥75; <80/≥80), baseline NIHSS (<10/≥10), time-to-intervention strata (>4.5-6, >6-9, wake-up), region (ANZ/Finland vs Taiwan), LVO status	NR (no software failure/unreadable rate reported)	Yes (clinical): higher mRS 0-1 at 90 d (adj RR 1.44); higher reperfusion ≥50%/≥90%; higher recanalization; more early neurologic improvement; workflow times not differentially reported (door-to-needle ≈2 h, no arm-wise effect)
[11]	Protocoling (stable chest pain/CAD diagnostic strategy)	Triage/selection (initial test choice)	CCTA (≥64-slice) vs ICA	None (no AI used)	ICA (initial invasive strategy)	Adjudicated clinical outcomes (MACE: CV death/MI/stroke); obstructive CAD by ICA per guidelines	Y; 26 centers in 16 countries	N/A (randomized strategy; no thresholding)	Y; prespecified subgroups reported as consistent (e.g., sex/center analyses)	~6% nondiagnostic CT	Y; earlier testing (median 3 vs 12 days), fewer major procedure-related complications (HR≈0.26), fewer revascularizations; MACE similar
[12]	AIS (suspected) - ED presentations	Score/Detect (automated ASPECTS; EIC detection)	NCCT (3-5 mm slices; Siemens scanners)	Commercial; Heuron ASPECTS v1.0.0.0 (deep-learning CNN)	Two stroke experts (consensus) as reference	Baseline expert consensus on NCCT (two experts); AIS confirmation by DWI used for cohort definition	N (single-center clinical validation; training at separate center)	Per-ROI thresholds chosen to achieve ≥90% specificity; dichotomized ASPECTS cut-offs (>4 vs ≤4; >6 vs ≤6)	N (region-level and dichotomized analyses only; no patient subgroups)	NR; no trial dropouts reported (noisy CTs excluded pre-enrollment)	N; no measured time/clinical outcome change, only potential decision support noted
[13]	AIS (anterior-circulation LVO)	Prognostic (predict final infarct volume & infarct growth rates)	CTP (native source images)	In-house deep-learning (deconvolution-free; no commercial name)	RAPID (iSchemaView) deconvolution/thresholding software	Final infarct volume delineated on follow-up imaging: MR CLEAN-NCCT day 5 (or 24 h); CRISP-MRI FLAIR day 5 (core-lab)	Y; independent external cohort (CRISP, multicenter)	Accuracy assessed by mean absolute difference (MAD) vs. ground truth; RAPID core rCBF < 30% (exploratory 38%) for reperfused and Tmax > 6 s for no-reperfusion; analyses stratified by reperfusion (mTICI)	Y; reperfusion strata (no/intermediate/2b-3), hypoperfusion intensity ratio (HIR) with threshold ≈0.36, target-mismatch profile	NR for AI; dataset exclusions for CTP quality: MR CLEAN 42/500 (8.4%), CRISP 2/190 (1.1%); manual AIF needed in 4 cases	N (no measured time or outcome change); authors propose “tissue clocks” for potential logistics optimization
[14]	Protocoling (CRT candidate risk stratification in HF)	Prognostic (12-mo death or HF hospitalization; all-cause mortality)	Clinical/EHR features (ECG, echo, demographics, comorbidities; no imaging)	In-house ML; Random Forest model (best of 6 tested)	Clinical stratifier: BBB morphology + QRS duration groupings	Trial-defined clinical outcomes from COMPANION (death; HF hospitalization)	N (validated on separate CRT-D arm within same multicenter trial; not an independent dataset)	Decision rule based on RF probability: quartiles for risk; fixed threshold ~0.76 reported for Se/Sp/PPV/NPV	Y - BBB/QRS subgroups vs RF quartiles; reclassification (e.g., LBBB ≥150 ms in worst RF quartile)	NR (not applicable; no unreadable/imaging failures reported)	N - No prospective workflow/clinical outcomes change; authors propose use to improve pre-implant shared decision-making
[15]	ICH/TBI	Detect (ICH + subtypes, calvarial fracture, midline shift, mass effect); Triage intent	NCCT (non-contrast head CT)	Commercial (Qure.ai); deep-learning head CT algorithms; version not specified in paper	Humans: (i) original clinical radiology reports (Qure25k) and (ii) three-radiologist consensus; individual radiologists compared on CQ500	Baseline consensus of 3 radiologists (CQ500)/NLP-parsed baseline radiology reports (Qure25k)	Y; external CQ500 dataset from 6 independent centres	Two preset operating points reported: high-sensitivity (~0.90 Sn) and high-specificity (~0.90 Sp) on ROC	Y - by finding (five ICH subtypes, calvarial fracture, midline shift, mass effect) and by dataset (Qure25k vs CQ500)	NR in manuscript (post-op/age<7/missing series were excluded a priori; no unreadable AI rate given)	N - No prospective workflow study; authors propose potential use to automate triage
[16]	AIS	Prognostic (90-day mRS prediction)	NCCT (acute-phase) + clinical variables	In-house research DL model (3D ResNet on NCCT + support-vector regression fused with clinical data); no commercial name/version stated	Software comparators: imaging-only DL model and clinical-only model; (MRI-based models referenced qualitatively)	90-day modified Rankin Scale from six multicenter trials/registries (DEFUSE2, DEFUSE3, CRISP, iCAS, Lausanne Univ. Hospital, Stanford Univ. Hospital)	N (six-fold cross-validation across 6 datasets; no held-out external cohort reported)	Regression MAE and ROC for unfavorable outcome (mRS > 2); AUC reported (0.91); no fixed Se/Sp operating threshold specified	Y - prespecified subgroups: sex, age bands, cohort/source dataset, CT timing (days from stroke to CT), treatment regimen (EVT, IV-tPA)	NR	N - retrospective modeling only; no workflow/clinical impact assessment reported
[17]	AIS	Prognostic (90-day mRS prediction)	MRI DWI (±B0), acquired 1-7 days post-stroke	In-house research DL (3D ResNet imaging model stacked with SVR clinical model; no commercial name/version)	Software comparators: clinical-only SVR (±24-h NIHSS), imaging-only DL; head-to-head within study	90-day modified Rankin Scale (mRS) assessed at ~90 days (range 60-120)	Y; external test on Lausanne University Hospital (n=280; single site)	Youden-index operating point for unfavorable outcome (mRS>2); AUC up to 0.92 (internal) and 0.90 (external); Se/Sp reported at Youden point	Y - analyses by treatment regimen (no tx/IV-tPA/EVT/both); additional tertile outcome analyses	NR	N - retrospective modeling; no workflow/clinical impact evaluation reported
[18]	Protocoling	Protocol selection & contrast decision (triage/protocoling)	Referral text for emergency brain MRI (no imaging input; predicts MRI protocol class ×12 and contrast use)	Mixed: In-house ML/DL models (Naive Bayes, SVM, XGBoost; FinBERT) and commercial LLM (OpenAI GPT-3.5 Turbo v0125; fine-tuned)	Humans: two emergency radiologists (non-neuroradiologists); Software: head-to-head vs ML (SVM/XGBoost/Naive Bayes) and FinBERT	Baseline consensus of two fellowship-trained neuroradiologists labeling protocol & contrast from referrals	N; single-institution retrospective dataset (Turku Univ. Hospital)	Fixed model accuracy on held-out test set; metrics: accuracy, (macro/weighted) F1, precision/recall; McNemar with Bonferroni; no ROC/AUC (probabilities not comparable)	Y - training-set size (100% vs 50% vs 50%+augmentation); per-class performance & confusion matrices; comparison to human readers	NR (no unreadables/failures reported after exclusions; n=1953 usable referrals)	N - no prospective workflow/clinical impact study; note that AI processed in seconds-minutes vs ~5-6 h human review (time estimates only)
[19]	AIS (stroke) detection	Detect (binary stroke vs normal)	NCCT (brain CT)	In-house CNN (“OzNet”); hybrid pipelines OzNet-mRMR-DT/kNN/LDA/NB/SVM (best: OzNet-mRMR-NB)	Software baselines: GoogLeNet, InceptionV3, MobileNetV2; classical ML (DT, kNN, LDA, NB, SVM). No human reader comparison	Labels from single-center dataset (Lady Reading Hospital, Pakistan): stroke vs normal per existing dataset annotations; no adjudication described	N; single institution; 10-fold internal CV only	No pre-specified fixed operating point; report accuracy/sensitivity/specificity/AUC under 10-fold CV (best pipeline accuracy 98.42%, AUC 0.99)	N (no patient/lesion subgroups; comparisons only across model variants)	NR (no unreadable/failed cases reported after exclusions)	N; retrospective methods paper-no prospective workflow or clinical impact evaluation
[20]	AIS	Detect (binary classification of MRI reports: AIS vs non-AIS)	MRI report text from conventional stroke MRI (T2, FLAIR, GRE, DWI, ADC, TOF-MRA)	In-house NLP/ML pipeline (R “quanteda”); classifiers: BLR, Naïve Bayes, Single Decision Tree (best), SVM	Software comparators (between ML models); no human reader comparator	Prospective AIS registry label (AIS defined by relevant MRI lesion + acute symptoms >24 h); non-AIS MRI reports as controls	N; single academic center (train/test split; 10-fold CV on training)	No pre-specified operating point; performance reported via F1-measure/accuracy/ROC (best SDT F1 = 93.2, accuracy = 98.0)	N (only sampling strategies/feature variants; no clinical subgroups)	NR (not reported)	N; retrospective method study-no workflow or clinical outcomes measured
[21]	TBI (severe)	Prognostic (6-mo mortality & unfavorable outcome [GOS 1-3])	NCCT (admission head CT)	In-house; customized CNN (AlexNet-based) imaging model + clinical LDA; ensemble “fusion” and “IMPACT-fusion”	Humans (3 attending neurosurgeons) and Software (IMPACT model); internal clinical vs imaging vs fusion models	Adjudicated 6-month Glasgow Outcome Scale by structured interview (3- or 12-mo substituted if 6-mo missing)	Y; TRACK-TBI external cohort (n=220) from 18 institutions	Fixed-specificity operating points (100%, 95%, 90%) to report sensitivity; ROC AUC primary metric	N (no patient subgroup analyses reported)	NR; cases with substantial motion artifacts excluded a priori	N; comparative performance vs neurosurgeons discussed, but no prospective workflow/clinical impact measured
[22]	AIS (post-EVT)	Detect (infarction detection)	DECT (Twin-Spiral); VNC & AI-optimized edema maps	In-house; device-specific “y” parameter derived with AI (SynthSR-based GM/WM segmentation) integrated into syngo.via edema-map post-processing	Software: conventional mixed DECT and default VNC (also contrasted with SECT conceptually)	Follow-up CT or MRI when available (n=32); otherwise baseline NCCT/CTP comparison (n=20)	N; single center (sites = 1)	Decision rule: ROI HU reduction on edema maps vs VNC/mixed; statistical comparison (p<0.001); no fixed Se/Sp threshold	Y; reconstruction “Resolution” smoothing levels 1/3/5 compared	0 reported (no motion-related image degradation noted)	N; only hypothesized benefits (earlier/clearer infarct visualization, differentiation from contrast staining)
[23]	AIS (LVO detection in suspected stroke)	Detect/Triage	Portable optical cerebral blood flow (speckle-contrast) monitor (forehead headset); bedside 70-s acquisition	Hybrid: Commercial hardware (Openwater optical CBF monitor) + in-house deep learning classifier (transformer; 5-fold CV)	Software/Usual care: prehospital stroke scales RACE (≥5) and LAMS (≥4)	CTA-defined anterior-circulation LVO (ICA/M1/M2/tandem) as reference standard	N; prospective two-center derivation (sites = 2)	Threshold chosen by Youden’s J; achieved Se 79% and Sp 84% (AUC 0.82)	Y; performance by Fitzpatrick skin types (1-3 vs 4-6)	16.7% (27/162 scans failed QC → 135 analyzable)	N; modeled reduction in false positives/negatives per 1000 encounters, but no measured workflow or outcomes impact
[24]	AIS	Score (ASPECTS)	NCCT (baseline); follow-up MRI used for reference	Commercial; RAPID ASPECTS v4.9 (iSchemaView)	Two neuroradiologists (human readers)	Consensus reference incorporating baseline NCCT, CTP, DSA, and follow-up MRI (3-5 days)	N; single institution (n=1)	Dichotomized ASPECTS ≥6 vs <6 reported; primary analyses via weighted κ agreement (no fixed Se/Sp)	Y; time from onset→imaging (<60, 60-<120, 120-<240, ≥240 min) and separate cohorts (LVO vs suspected no-LVO)	~20% not processable by software (22 in cohort 1; 10 in cohort 2)	N; no prospective workflow/time metrics. Compute time ~2-4 min noted; hypothetical impact: 7/100 would have been excluded from thrombectomy by software criteria.
[25]	AIS	Segment (core lesion on DWI)	MRI (DWI + ADC; adjunct FLAIR for outlining)	In-house research algorithm; ATLAS (Automatic Tree Learning Anomaly Segmentation)	Software baselines: COMBAT Stroke and generalized thresholding; human expert outlines used only as reference	Expert manual DWI lesion outlines (with ADC/FLAIR to avoid T2 shine-through)	N (multicenter internal LOO cross-validation within I-Know; ~4 sites: Aarhus, Hamburg, Lyon, Girona)	Post-processing probability threshold = 0.25 after Gaussian smoothing; tree pruning depth chosen by CV AUC; node splits by max Youden’s J	N (no prespecified clinical subgroups; method comparisons only)	Not reported (no unreadable cases stated)	N (methodology/performance study; no time or clinical pathway effects measured)
[26]	AIS	Score/Detect early infarct signs via ASPECTS	NCCT	Commercial: e-ASPECTS (Brainomix®, Oxford, UK)	Humans: three neuroradiologists (B, C, D)	Definite infarct core after best medical care (thrombolysis and/or thrombectomy) used as reference	N (single-center; 1 site)	Not specified (software default, region-based ASPECTS; no fixed Se/Sp thresholding reported)	Y: stratified by patient groups-(I) normal brain, (II) leukoencephalopathy, (III) prior infarcts, (IV) atypical parenchymal defects	Not reported	N (no time or pathway outcomes assessed; interpretive support only)
[27]	AIS	Score/Detect early infarct signs via ASPECTS	NCCT (NECT; reconstructions FBP, IR2, IR4)	Commercial: e-ASPECTS (Brainomix, Oxford, UK)	Humans: 4 blinded readers (2 residents, 2 consultants)	Expert-defined reference using clinical info, baseline multimodal CT/CTP, and follow-up scans	N (single center; n=1 site)	None specified; performance assessed by weighted κ and ICC across reconstructions (no fixed Se/Sp threshold)	Y: by CT reconstruction level (FBP vs IR2 vs IR4) and reader experience (residents vs consultants)	NR	N (methodological/diagnostic agreement study; no reported pathway or clinical outcome impact)
[28]	FFT	Detect (differentiate FFT vs carotid plaque)	CTA	In-house quantitative shape analysis (MaZda-derived shape descriptors) with classifiers (logistic regression, LDA, ANN, SVM)	None (no human-reader comparison)	Follow-up imaging outcome: resolution/decrease on CTA after anticoagulation/antiplatelet = FFT; unchanged = plaque	N (single center; n=1 site)	ROC-derived threshold for combined 5-feature logistic regression: Se 87.5%, Sp 71.4%, AUC 0.85 ± 0.09; cross-validation (LOO/10-fold) reported	N	NR (no unreadable cases reported)	N (exploratory diagnostic feasibility; no workflow/outcome effects assessed)

Discussion

Across 20 studies spanning North America, Europe, and Asia-Pacific, we found a broad and heterogeneous evidence base for imaging-AI in emergency neuroimaging pathways that encompasses ischemic stroke, ICH, and TBI, with smaller but notable contributions in protocoling/triage and prognostic modeling. Consistent with the operational reality of acute care, CT-based modalities predominated, while MRI was leveraged for core delineation and outcome prediction. Diagnostic performance was generally strong for NCCT hemorrhage/fracture detection and competitive for automated ASPECTS at the case level, albeit with region-level sensitivity constraints; prognostic fusion models reached AUCs ≈ 0.90-0.92. Evidence for workflow and clinical impact was more limited but directionally encouraging; AI-guided selection or validated clinical algorithms reduced unnecessary imaging and, when imaging directly informed eligibility, improved functional outcomes. Collectively, these findings align with the maxim that accuracy is necessary but not sufficient: measurable patient benefit most clearly emerged when AI outputs were operationalized to influence time-critical decisions.

Two task clusters consistently demonstrated high discriminatory performance. First, multicenter NCCT systems for hemorrhage detection reached balanced sensitivity and specificity in the 80-90% range with AUCs ~0.92-0.97 across ICH subtypes and related targets (e.g., midline shift, mass effect), as exemplified by the Qure.ai ensemble [15]. These results closely mirror high-throughput challenge and external-validation work: Wang et al. reported AUCs of 0.988 for “any ICH” on RSNA test data and 0.949-0.964 on two independent datasets, with sensitivity and specificity of ≈0.95 and ≈0.94, respectively, and robust subtype-level performance [29]. Smaller engineering-led series also corroborate strong detection, with Lewick et al. achieving an overall area under the receiver operating characteristic curve (AUROC) of 0.985 and tunable precision-recall trade-offs [30], and Majumdar et al. demonstrating 98% case-level specificity alongside lesion-level sensitivity of 81% after post-processing to suppress bone-adjacent false positives [31]. A teaching-hospital study from Malaysia similarly reported high sensitivity (0.97) and specificity (0.93) on a 200-image dataset [32]. While these latter studies are limited by dataset size or selection, their operating points reinforce the feasibility of safety-first deployment (i.e., recall-favoring thresholds) with manageable false positives.

Second, prognostic fusion models that integrate imaging with clinical variables performed well. In ischemic stroke, fused ResNet-SVR models using one- to seven-day NCCT or DWI achieved AUC ≈ 0.90-0.92 for unfavorable 90-day mRS both internally and on external cohorts [16,17]. In severe TBI, a CT-plus-clinical stacking ensemble achieved AUC 0.92 for six-month mortality at fixed 90% specificity [21]. These figures exceed or match many guideline or score-based baselines and suggest practical triage value for neurocritical care.

Other tasks showed good but more modest accuracy. A shape-analysis approach on CTA distinguished carotid free-floating thrombus (FFT) from plaque with an AUC ~0.85 [28]. Automated ASPECTS tools demonstrated high specificity but moderate sensitivity at the region level, e.g., Heuron ASPECTS with region-level AUC 0.88 (specificity ~97%, sensitivity ~63%) [12], and e-ASPECTS with case-level sensitivity/specificity of 83%/57% against follow-up core [26]. Studies of diffusion-lesion segmentation (e.g., ATLAS) reported improved overlap metrics over classical thresholding without standard diagnostic endpoints [25], and a post-EVT DECT post-processing method showed qualitative enhancement rather than quantified accuracy [22]. Emerging NLP for ED brain-MRI protocol achieved 84% protocol accuracy and 91% contrast decision accuracy in an external test set [18].

Three methodological choices influenced apparent performance. One was accuracy against expert consensus on baseline imaging (common in ASPECTS studies) yielded different operating points than accuracy against follow-up infarct core or trial-adjudicated outcomes, e.g., e-ASPECTS sensitivity fell when benchmarked to follow-up core rather than baseline reads [26]. In contrast, prognostic models benefited from hard outcome anchors (mRS, Glasgow Outcome Scale (GOS)), improving the interpretability of AUCs [17,21]. Per-region vs. per-patient endpoints drive different sensitivity/specificity trade-offs; region-level detection is inherently harder due to small and low-contrast cortical territories early after onset [12]. Performance depended on reconstruction strength and slice thickness; Wang et al. explicitly modeled slice thickness and demonstrated robust generalization across 0.6-5 mm data [29]. Iterative reconstruction can improve conspicuity and consistency in ASPECTS-type tasks [27], and DECT variants modify contrast-to-noise [22], further underscoring the need for cross-vendor validation.

Our synthesis is broadly concordant with large external-validation and challenge reports that place “any ICH” AUCs near 0.95-0.99 and sensitivity/specificity in the mid-90s [29]. However, some engineering papers reported exceptionally high accuracies on small, curated sets (e.g., 0.95 accuracy on n=200 in paper) [32], which likely overstate real-world performance relative to our multicenter clinical cohorts [15]. For ASPECTS, our finding of high specificity but only moderate region-level sensitivity diverges from newer, large-scale DL systems that report overall-level AUC ≈0.85 and sensitivity ~0.95 for ASPECTS≥6 with substantial human-AI agreement and marked time savings [33,34]. This discrepancy is explained by differences in labels (CTP- or expert-anchored ASPECTS vs. follow-up core), sample size, and explicit human-in-the-loop optimization in the latter studies. Finally, our inclusion of FFT classification (AUC ~0.85) reflects a niche but clinically consequential CTA task less represented in broad ICH/stroke AI reviews.

Two studies quantified meaningful workflow shifts. In pediatrics, using a validated clinical prediction rule reduced CT use by ~10 percentage points and increased clinical clearance by ~16 percentage points without reported safety signals [9]. In a cardiology pathway included here to illustrate imaging-guided workflow design, initial CT shortened time-to-testing (median 3 vs. 12 days; HR 1.54) and reduced major procedure-related complications compared with an invasive-first strategy [11]. Although not strictly AI, this trial demonstrates how an imaging-first, standardized strategy can accelerate care and mitigate iatrogenic risk, principles directly relevant to AI-assisted triage and protocoling. Most neuroimaging AI studies optimized offline discrimination without embedding prospective operational interventions (e.g., prioritized worklists, automated alerts, protocol selection integrated into ordering). Consequently, classic time metrics (DTN, door-to-groin, time-to-report) were rarely measured. This evidence gap is particularly notable because stroke outcomes are tightly time-dependent.

High-quality, non-AI literature demonstrates clear links between process time and outcomes. In a 61,426-patient cohort of tissue plasminogen activator (tPA)-treated AIS, each 15-minute delay in DTN within the first 90 minutes independently increased one-year mortality (HR 1.04) and readmission (HR 1.02) [35]. A national quality-improvement initiative (Target: Stroke) nearly doubled the proportion of patients treated within 60 minutes and was associated with lower in-hospital mortality, fewer symptomatic ICH events, and higher home discharge [36]. The contrast is instructive: our corpus shows that AI can reach high diagnostic accuracy, but unless the tools are engineered to directly compress the clock or reduce unnecessary steps, measurable time gains remain elusive. Emerging ASPECTS systems begin to bridge this gap by cutting per-case assessment times from ~2 minutes for physicians to ~33 seconds for AI, with substantial efficiency gains when humans are assisted [34].

Only one randomized trial in our set directly linked imaging-guided selection to better functional outcomes: perfusion-mismatch-selected alteplase improved 90-day functional independence (mRS 0-2) without a mortality difference [10]. Prognostic models in stroke and TBI showed strong discrimination for 90-day mRS and six-month mortality, respectively, but were not tested as interventional decision aids [16,17,21]. The mechanistic plausibility that AI-enabled workflow gains would improve outcomes is supported indirectly by the association between shorter treatment times and lower mortality/readmission [35] and by improved in-hospital outcomes following systematic DTN reduction [36]. While these are not AI trials, they quantify the outcome elasticity to time, implying that even modest AI-driven time reductions (e.g., faster ASPECTS determination, earlier hemorrhage identification, faster protocol selection) could translate to outcome benefits if embedded within streamlined pathways. Heetderks’ population-level analysis of ED headache visits highlights that broad CT use did not “rule out” impending stroke [37]; many patients imaged for headache returned with stroke within days, reflecting the limited sensitivity of CT for early ischemia [37]. This underscores two points: detection tasks should be aligned with modality strengths (CT excels at hemorrhage; early ischemia is subtle), and AI must be integrated with clinical pathways (observation, follow-up imaging, and decision rules) to mitigate false reassurance.

The evidence is denser for stroke than for trauma. Trauma contributions in our corpus focus on head CT detection and TBI prognostics [15,21]. Pediatric trauma demonstrates that algorithmic triage (clinical prediction rules) can safely reduce CT use at scale [9]. Gaps remain for comprehensive AI-assisted decision support in polytrauma and for pediatric neuroimaging AI calibrated to age-specific physiology. CT dominates detection/triage (ICH, fracture, early ischemic change), while MRI (particularly DWI) supports core segmentation and outcome prediction [17,25]. For ASPECTS, CT-based automation shows improving accuracy and reliability, and quantitative attenuation-based approaches on NCCT/CTA offer an interpretable alternative with similar or better correlation to core volume [38]. These findings align with modality physics: blood products are highly conspicuous on NCCT; cytotoxic edema can be subtle on NCCT early but conspicuous on DWI. Commercial tools feature prominently in ASPECTS and perfusion processing (RAPID; Brainomix; Heuron), whereas in-house models are common in segmentation and prognostics [17,21,25]. External validation is uneven: hemorrhage detection and ASPECTS systems increasingly report multi-site testing and prospective uptake [29,34], but many models remain single-center. Differences in reference standards (expert consensus vs. follow-up core), thresholds, case mix, scanner vendors, and reconstruction algorithms create substantial heterogeneity [39-42]. Notably, studies that specified reconstruction details or modeled slice thickness [29,40,43] reported stronger generalization.

Multicenter training/validation and cross-vendor robustness testing improved transportability [15,29,34,44-46]. However, many included studies were single-site validations, and some used retrospective convenience cohorts with spectrum effects. Implementation studies that prospectively monitor site-level performance are needed. To convert accuracy into clinical impact, organizations should ensure tight integration across the Picture Archiving and Communication System (PACS), Radiology Information System (RIS), and Electronic Health Record (EHR) for worklist prioritization and alerting; measure alert latency and time to report; and establish governance processes for quality assurance (QA), model-drift monitoring, and escalation rules. The experience from Target: Stroke shows that standardizing processes at scale, with decision support and feedback loops, can shift outcomes; AI initiatives should emulate this infrastructure [36,47,48]. Saliency and error-pattern analyses can reduce false positives [29,31] and inform threshold setting [30]. Given CT’s limited sensitivity for hyperacute ischemia [37], pathway safeguards (observation, MRI triggers, repeat imaging) should be encoded. For ASPECTS, combined human-AI reading improved both accuracy and speed and maintained consensus alignment [33,34]. Several datasets were adult-dominant; pediatric trauma evidence came from a North American network [9,49,50]. Resource-limited settings may benefit most from automated ICH detection/triage [32], but external validation and language-adapted NLP [18] are essential. Procurement should require subgroup reporting (age, sex, scanner vendor, slice thickness) and fairness audits.

Near-term deployment should include high-yield detection - adopt NCCT hemorrhage/fracture detection tools that demonstrate multicenter validation and tunable thresholds, deploying them as triage/second-read with human oversight [15,29,30,31,32]. Implement automated ASPECTS to standardize scoring and accelerate downstream decisions; prioritize systems with prospective use data and demonstrated reader-assistance benefits [33,34]. Use fused imaging-clinical models for risk stratification and shared decision-making in severe TBI and AIS, with the caveat that prospective management studies are pending [16,17,21]. Couple accuracy deployments with prospective workflow trials that capture DTN/door-to-groin/time-to-report, and favor end points tied to functional outcomes (mRS/GOS). The magnitude of benefit observed in time-to-treatment literature [35,36,51] indicates that even modest, sustained time savings could be clinically meaningful. Favor vendors with external validation, transparent reference standards (e.g., follow-up core vs. baseline consensus), and in-product monitoring (calibration plots, sensitivity/specificity at site). Require drift detection, slice-thickness robustness, and cross-reconstruction checks [29]. Embed escalation rules for discordant AI-human reads and define safety nets for CT-negative but clinically suspicious ischemia [37].

The included body of evidence and our synthesis have several limitations. Study-level heterogeneity is substantial in populations, scanners, reconstruction parameters, and ground truths. Randomized or quasi-experimental studies of AI-in-workflow are scarce; two randomized trials in our tables concern imaging strategy or perfusion-guided thrombolysis rather than AI per se [10,11]. Head-to-head AI vs. no-AI implementation studies were limited, and some cohorts (e.g., cardiology; post-EVT imaging) were included for conceptual completeness rather than direct ED applicability. Reporting bias toward positive AUCs is possible, particularly in smaller or engineering-focused studies [30-32]. Finally, our synthesis is constrained by English-language sources and by inconsistent reporting of key metrics (e.g., alert latency, time-to-report).

Future work should prioritize pragmatic pathway trials, ideally cluster-randomized or stepped-wedge designs, that embed AI directly into ED stroke and TBI workflows with prespecified process and patient end points such as DTN, door-to-groin, time-to-report, 90-day mRS, and six-month GOS; the strong time outcome relationship shown by Man et al. (2020) and the system-level gains from Target [35], stroke justify outcome-powered evaluations [36]. Cluster and stepped-wedge designs are particularly appropriate because once AI-driven worklists or alerts are switched on, contamination across clinicians and shifts is unavoidable, and secular changes in staffing, imaging capacity, or parallel quality-improvement initiatives can otherwise bias time-based outcomes, so time metrics should be analysed with adjustment for background operational factors (e.g., time-of-day, weekday vs. weekend, ED crowding indices, scanner downtime, and co-implemented pathway changes) to attribute effects more credibly to the AI intervention rather than to global system changes. Beyond discrimination and accuracy, studies must report calibration, temporal and external validation, decision-curve net benefit, and cost-effectiveness at clinically realistic thresholds using local resource and salary data, and should prespecify continuous performance monitoring with site-level dashboards, including drift detection and explicit thresholds and governance processes for pausing, rolling back, or recalibrating models. Site-specific recalibration at go-live should include both global intercept/slope updates and, where needed, subgroup-specific recalibration for key patient strata (e.g., age, sex, race/ethnicity, stroke vs. TBI, comorbidity burden) and stratified performance across scanner vendors and reconstruction protocols [30].

Robustness and equity require cross-vendor and cross-reconstruction testing with explicit modelling of slice thickness [29], subgroup analyses by age, sex, and race/ethnicity, including pediatric vs. adult cohorts, and fairness audits for both detection and ASPECTS scoring, with external validation in resource-limited settings [32]. Where possible, these subgroup and fairness analyses should be prospectively specified and, for high-stakes tasks, powered to detect clinically important differences in both performance and outcomes across demographic and clinical strata. Integration science should quantify alert latency, worklist prioritization efficacy, reader workload, and escalation protocols, and test combined human-AI reading paradigms that have been shown to accelerate and standardize ASPECTS without sacrificing accuracy [33,34]; given CT’s limits for hyperacute ischemia, structured observation and MRI-trigger rules should be prospectively evaluated to avoid false reassurance [37]. Finally, target selection and triage merit focused development, including AI-guided prehospital tools such as portable sensors for LVO, which already show promising discrimination, and AI-assisted CTA interpretation to optimize EVT selection, building on early successes like shape-based classification of carotid FFT [23,28].

## Conclusions

AI-assisted CT and MRI are ready to augment, but not replace, acute stroke and trauma care. Across 20 studies, tools for NCCT hemorrhage/fracture detection and automated ASPECTS consistently delivered strong discrimination, and fused imaging-clinical models showed high prognostic accuracy. However, evidence connecting these gains to faster treatment and improved patient outcomes remains sparse; benefits were most apparent when AI outputs directly informed eligibility or simplified time-critical decisions. Signals include reduced pediatric CT use and improved functional outcomes when imaging selection guided thrombolysis, but randomized, workflow-embedded AI trials are rare. To convert accuracy into impact, deployment must be coupled to pathway engineering: tight PACS/RIS/EHR integration, measured alert latency and time-to-report, prioritized worklists, and explicit escalation rules for discordant reads and CT-negative but clinically suspicious ischemia. Robustness, calibration, and equity are also non-negotiable, with cross-vendor and cross-reconstruction testing, per-site recalibration, and subgroup/fairness audits. Policy and procurement should favor externally validated systems with transparent reference standards and in-product monitoring, and require post-market performance surveillance. Clinically, near-term priorities include hemorrhage/fracture triage, standardized ASPECTS assistance, and risk stratification in severe TBI and ischemic stroke, implemented as human-AI collaboration. However, translating this apparent “AI readiness” into routine benefit will depend on pragmatic implementation barriers being addressed, including clinician trust and adoption, interoperability across heterogeneous PACS/EHR and vendor ecosystems, and compliance with evolving regulatory and data-governance frameworks. Given the substantial and heterogeneous costs of acute stroke and trauma care, future work should also quantify the cost-effectiveness and budget impact of imaging-AI deployments across different health-system settings, particularly in resource-constrained environments. Finally, outcome-powered, pragmatic, cluster, or stepped-wedge trials should quantify effects on DTN, door-to-groin, time-to-report, 90-day mRS, and six-month GOS across systems.
